# Genome‐Wide Blood DNA Methylation Profiling in Birch Pollen Allergic Patients Undergoing Allergen‐Specific Immunotherapy

**DOI:** 10.1111/all.70094

**Published:** 2025-10-09

**Authors:** Angelika Lahnsteiner, Victoria Ellmer, Mengzhen Hao, Line Kring Tannert, Serge A. Versteeg, Carsten Bindslev‐Jensen, Ronald van Ree, Angela Risch, Lorenz Aglas

**Affiliations:** ^1^ Department of Biosciences and Medical Biology Paris Lodron University of Salzburg Salzburg Austria; ^2^ Center for Tumor Biology and Immunology (CTBI) Paris Lodron University of Salzburg Salzburg Austria; ^3^ Odense Research Center for Anaphylaxis Odense University Hospital Odense Denmark; ^4^ Department of Experimental Immunology Amsterdam University Medical Centers Amsterdam the Netherlands; ^5^ Department of Dermatology and Allergy Center Odense University Hospital Odense Denmark; ^6^ Department of Otorhinolaryngology Amsterdam University Medical Centers Amsterdam the Netherlands; ^7^ Institute of Pathophysiology and Allergy Research, Center for Pathophysiology, Infectiology and Immunology Medical University of Vienna Vienna Austria

**Keywords:** allergen‐specific immunotherapy, birch pollen allergy, CD8+ T cells, DNA methylation, epigenome‐wide DNA methylation study, HLA, NK cells

## Abstract

**Background:**

Until now, no study has investigated the impact of allergen immunotherapy (AIT) on genome‐wide DNA methylation in a longitudinal set‐up. Herein, we investigated whether differences in DNA methylation occur in birch pollen allergic patients undergoing 6 months of birch pollen AIT, assessed alterations in methylation‐based blood cell type composition, and correlated DNA methylation to serological AIT biomarkers.

**Methods:**

We performed genome‐wide DNA‐methylation analysis on bisulfite‐converted DNA derived from whole blood samples of 16 birch pollen‐allergic patients (pre–/post‐birch pollen AIT) and 15 placebo (pre‐/post‐placebo treatment).

**Results:**

Our analysis identified cg22187251, located within a regulatory region upstream of the glucosaminyl (N‐acetyl) transferase 2 (*GCNT2*) gene and cg22336863 upstream of the transcription start site of actin binding rho activating protein (*ABRA*), as hypermethylated. Functional assays revealed that these regions exhibit methylation‐dependent promoter and enhancer activities. We identified differentially methylated positions within the HLA gene complex, and an AIT‐specific increase of CD8+ T cell populations accompanied by a decrease in natural killer (NK) cell proportion. Strong to moderate correlations with clinical biomarkers (such as specific IgG_4_) were observed for 42% of the top 100 differentially methylated positions.

**Conclusion:**

*GCNT2* and *ABRA* are implicated in Rho‐signaling, a pathway involved in Th2 differentiation. GCNT2 modulates the SMAD‐dependent TGF‐β pathway, indicating a role in mediating AIT‐induced immunotolerance. This is the first longitudinal study investigating DNA methylation changes induced by birch pollen AIT.

## Graphical abstract

Whole genome‐wide DNA methylation analysis of whole blood of patients undergoing 6 months of birch pollen subcutaneous allergen immunotherapy revealed epigenetic changes linked to allergen immunotherapy. Birch pollen AIT decreases methylation in HLA genes and induces methylation in genes associated with Th2 and TGF‐β pathways, increases CD8+ T cell population and decreases NK cells. 42% of the top 100 differentially methylated positions correlate with AIT serum biomarkers (e.g., IgG4). ABRA, actin binding rho activating protein; AIT, allergen immunotherapy; BPE, birch pollen extract; GCNT2, glucosaminyl (N‐Acetyl) transferase 2; HLA, human leukocyte antigen; NK, natural killer cell; TGF‐β, transforming growth factor‐beta; Th, T helper cell.

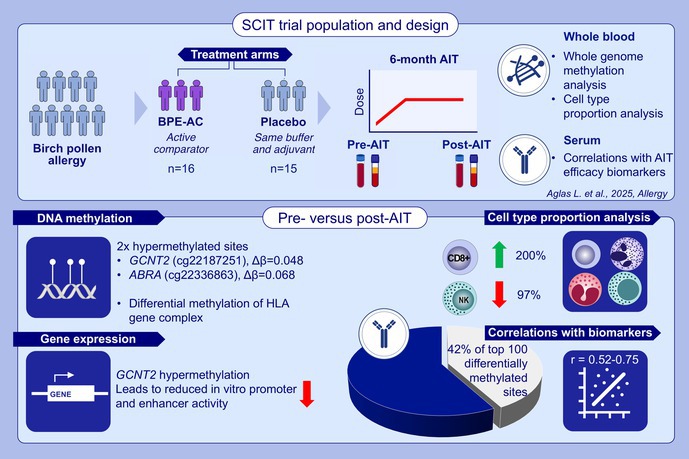

## 1 Introduction

Worldwide, more than 400 million people are affected by allergic rhinitis, or asthma caused by pollen exposure [1]. Birch pollen are among the most prevalent types of pollen found in Northern and Central Europe [2, 3]. Allergic symptoms occurring during the pollen season represent a major burden for the patients and have a high impact on their quality of life. Both genetic and epigenetic factors contribute to allergy development. While single nucleotide polymorphisms (SNPs) represent variations in the DNA sequence, epigenetic modifications such as DNA methylation are environmentally influenced changes that regulate gene expression without altering the genetic code. DNA methylation typically involves the addition of a methyl group to cytosine residues at CpG dinucleotides.

Several genome‐wide (GWAS) and epigenome‐wide association studies (EWAS) have been performed within the framework of allergic diseases to understand the underlying immunological mechanisms driving the disease [4, 5, 6, 7]. These studies identified risk SNPs, that is, genomic loci associated with an increased likelihood of developing allergies, or differentially methylated regions in genes linked to immune functions. Ek et al. identified 15 CpG sites associated with IgE levels [8], while Ferreira et al. reported 19 risk SNPs linked to asthma and hay fever [9]. Recent EWAS have shown that DNA methylation is altered in affected compared to healthy individuals in childhood asthma [10], house dust mite allergy [11], cow's milk allergy [12] and birch pollen allergy [13].

Currently, the only treatment promising long‐term or even lifelong tolerance is allergen‐specific immunotherapy (AIT) involving a gradually increased administration of the allergen over 3–5 years to induce tolerance via the induction of regulatory T and B cells and of IgG antibodies blocking the IgE response [14]. Up to now, no epigenetic study has investigated the impact of AIT on DNA methylation in a longitudinal design leveraging the benefits of paired analyses. Therefore, the objective of our study was (i) to investigate differences in DNA methylation pre‐ versus post‐birch pollen AIT in comparison to placebo‐treated patients using a longitudinal study design, (ii) to perform a methylation‐based assessment of cell type proportions altered in the course of AIT, and (iii) to investigate correlations between DNA methylation and serological AIT biomarkers. Here we report on the first pilot study demonstrating the potential of DNA methylation analysis after AIT to monitor treatment progression.

## 2 Methods

### 2.1 Study Design

The randomized, placebo‐controlled subcutaneous allergen immunotherapy (SCIT) trial was conducted with Alutard SQ ( *Betula verrucosa* , ALK, Horsholm, Denmark, maintenance dose 100.000 SQ/ml) as established birch pollen extract‐based SCIT vaccine (termed BPE‐SCIT in the following) with market authorization [14]. The SCIT trial was performed from September 2018 to March 2019 at the Allergy Center, Odense University Hospital, Denmark (ClinicalTrials.gov Identifier: NCT04912076). Adult birch pollen allergic patients with moderate to severe allergic rhinitis and rhinoconjunctivitis symptoms (with or without concomitant mild to moderate persistent asthma) were enrolled according to clearly defined inclusion and exclusion criteria, such as clinical history of allergic rhinitis, a positive skin prick test (mean wheal diameter ≥ 3 mm), and specific IgE against the birch pollen extract (≥ 0.7 kU/L, Table 1). Duration of rhinoconjunctivitis symptoms ranged between 2.8 and 59.77 years; 5 of 31 (16.1%) were diagnosed with asthma and 3 of 31 (9.7%) with atopic dermatitis. Allergic symptoms other than against birch pollen were reported for grass pollen, house dust mites, animal dander, ragweed, and molds in 61.3%, 3.2%, 16.1%, 3.2%, and 3.2% of patients, respectively. For details on the clinical trial refer to Aglas et al. [15]. Sixteen patients were treated with BPE‐SCIT, while 15 patients received placebo (adjuvant aluminum hydroxide in the same concentration as in Alutard SQ). The study was approved by the Danish Medicines Agency and the Regional Committees on Health Research Ethics for Southern Denmark (EudraCT number 2018‐001486‐17). Informed consent was signed by all patients.

**TABLE 1:** Demographic data and serum biomarker data (IgG levels and serum inhibitory activity) used as proxy for AIT efficacy.

	BPE‐SCIT	Placebo
	*n* = 16	*n* = 15
Gender (female/male)	8/8 (50% female)	9/6 (60% female)
Age (years)	38.27 ± 15.2	32.07 ± 32.07
BPE specific IgE (kU/L)		
Pre‐treatment	9.12 ± 13.20	14.8 ± 9.0
Post‐treatment	10.36 ± 11.03	12.8 ± 8.7
Bet v 1 specific IgE (kU/L)		
Pre‐treatment	9.59 ± 13.97	15.3 ± 10.8
Post‐treatment	9.25 ± 10.36	12.1 ± 9.0
BPE specific IgG_1_ [kU/L]		
Pre‐treatment	0.18 ± 0.1	0.18 ± 0.06
Post‐treatment	0.69 ± 0.35	0.17 ± 0.05
Bet v 1 specific IgG_1_ [kU/L]		
Pre‐treatment	0.09 ± 0.05	0.08 ± 0.04
Post‐treatment	0.47 ± 0.26	0.07 ± 0.04
BPE specific IgG_4_ [kU/L]		
Pre‐treatment	224.59 ± 322.33	195.58 ± 203.91
Post‐treatment	2476.34 ± 3091.51	168.57 ± 184.11
Bet v 1 specific IgG_4_ [kU/L]		
Pre‐treatment	139.11 ± 204.31	119.32 ± 142.6
Post‐treatment	1920.05 ± 1916.78	116.08 ± 145.5
BPE specific IgG [kU/L]		
Pre‐treatment	0.56 ± 0.26	0.45 ± 0.19
Post‐treatment	2.98 ± 1.5	0.49 ± 0.22
Bet v 1 specific IgG [kU/L]		
Pre‐treatment	0.57 ± 0.3	0.42 ± 0.18
Post‐treatment	1.6 ± 0.69	0.28 ± 0.17
BPE specific IgG_4_/IgG_1_ ratio		
Pre‐treatment	1.18 ± 1.65	1.08 ± 1.06
Post‐treatment	4.3 ± 5.99	1.06 ± 0.95
Bet v 1 specific IgG_4_/IgG_1_ ratio		
Pre‐treatment	2.02 ± 3.69	1.36 ± 1.33
Post‐treatment	5.86 ± 8.55	1.39 ± 1.41
Inhibition ELISA^a^		
Pre‐treatment	12.58 ± 11.78	3.7 ± 9.96
Post‐treatment	36.12 ± 20.72	8.68 ± 10.12
Inhibition mediator release assay^a^		
Pre‐treatment	−7.07 ± 40.3	0.13 ± 34.35
Post‐treatment	44.26 ± 37.29	−2.17 ± 37.23
Facilitated antigen binding inhibition assay^a^		
Pre‐treatment	49.08 ± 37.05	44.03 ± 23.49
Post‐treatment	98.25 ± 2.77	27.01 ± 26.66
Facilitated antigen binding competition assay^a^		
Pre‐treatment	31.08 ± 28.29	30.69 ± 21.26
Post‐treatment	88.52 ± 13.58	21.64 ± 16.79

*Note:* Data are expressed as mean ± standard deviation (SD).

^a^ Data are expressed as percentage inhibition relative to non‐inhibited values.

**FIGURE 1:**
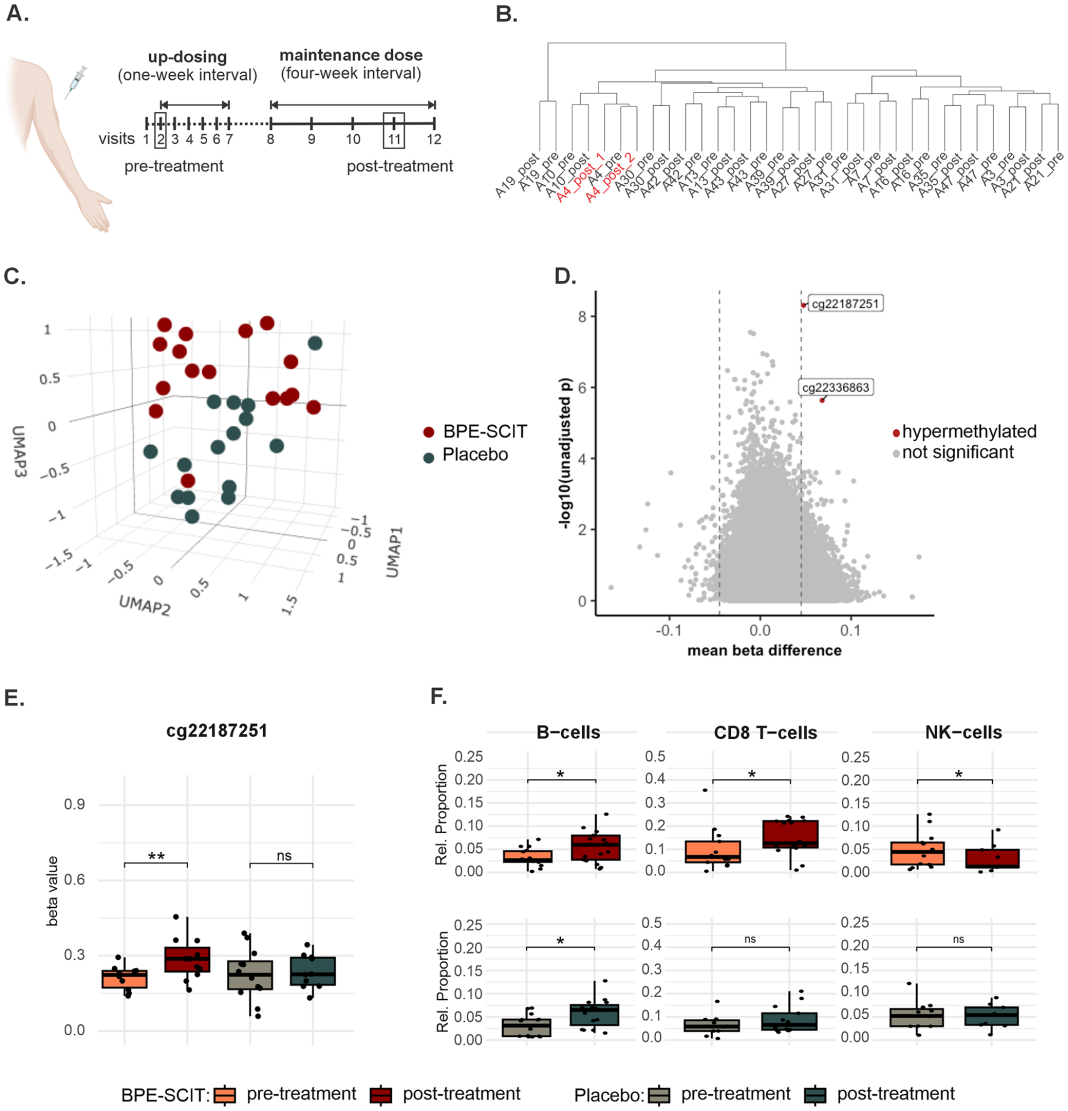
Comparison of DNA‐methylation in birch pollen allergic patients before and after birch pollen extract‐based subcutaneous‐ allergen immunotherapy (BPE‐SCIT). (A) Study design of the clinical trial. Genome‐wide DNA‐methylation was compared between samples collected at visit 2 (pre‐treatment) and visit 11 (post‐treatment). The figure was created with bioRender.com. (B) Hierarchical clustering of samples using Manhattan distance. Replicates labeled in red cluster together in one branch, demonstrating reproducibility. (C) Significant sample clustering (*p* = 0.031) was detected using uniform manifold approximation and projection (UMAP) analysis based on Δβ between pre‐ and post‐treatment of the top 100 differentially methylated sites after BPE‐SCIT treatment. (D) Volcano plot showing the two top significant differentially methylated sites in cg22187251 (*GCNT2*) and cg22386863 (*ABRA*). The *y*‐axis represents the −log10 unadjusted *p*‐value. The dashed line indicates the mean beta difference of 0.045. (E) Boxplots of cg22187251 comparing DNA methylation of pre‐ and post‐treated. *p*‐values are false discovery rate (FDR)‐corrected. (F) Cell type composition was determined by 50,000 most variable sites based on a reference methylome (22) for BPE‐SCIT (upper panel) and placebo (lower panel) in pre‐ and post‐treated samples. *p < 0.05, **p < 0.01.

Whole blood samples were collected from the patients at ambulatory visit 2, that is, before the initial SCIT administration, termed pre‐treatment in the following, and at visit 11, that is, at the end of the 6‐month clinical trial (post‐treatment; Figure 1A).

### 2.2 DNA Methylation Analysis With Illumina Infinium MethylationEPICv1 Arrays

DNA was extracted from whole blood of 32 samples (16 paired samples) treated with BPE‐SCIT and 30 placebo samples (15 paired samples) with the ReliaPrep Blood gDNA MiniPrep System (Promega, Austria) according to the manufacturer's instructions, but without vortexing to avoid DNA fragmentation. Bisulfite conversion and Infinium MethylationEPICv1 arrays (850 k, Illumina, Austria), covering approximately 850,000 CpG sites across the whole genome, were performed at Diagenode (Belgium).

Data analysis was conducted via the RnBeads 2.0 pipeline [16] in R version 4.4.1 [17] and RStudio [18]. A detailed explanation about the packages used during preprocessing, normalization, and differential methylation calling can be found in the Supplement Methods Section 1.1.

Differentially methylated positions (DMPs) were called by using a paired comparison of each sample pair (pre‐ and post‐treatment) applying the empirical Bayes method ‘limma’ [19] and identified as statistically significant with a false discovery rate (FDR‐) corrected *p* < 0.05. To avoid the detection of false positives, we applied a threshold requiring an absolute mean beta difference of |Δβ| > 0.045, where β represents the DNA methylation level, ranging from 0 (unmethylated) to 1 (fully methylated). In addition, two technical replicates were included, one for BPE‐SCIT sample A4 and one for placebo sample P1, to evaluate reproducibility.

### 2.3 Hierarchical Clustering of DNA Methylation Data

Clustering of DNA methylation data for each sample was performed with the hclust function using Manhattan distance [20].

### 2.4 Dimensionality Reduction

For simple exploratory variance analysis, a principal component analysis (PCA) was performed on Δβ values of post‐ versus pre‐treatment using the top 100 DMPs sorted by the *p*‐value with the two highest Eigenvalues for PC1 and PC2, capturing 26% of global variance. Clustering of patients according to quadrant Q2 and Q3 was further analyzed by calculating the difference in distribution using a two‐tailed Mann Whitney test. Following PCA, uniform manifold approximation and projection (UMAP) analysis was performed with the same dataset. Detailed information is given in Supplement Methods Section 1.2.

### 2.5 Linear Regression Model to Evaluate Cell Type Specific Methylation Changes of *GCNT2*–cg22187251

To evaluate the association of cg22187251 DNA methylation with different cell types as well as timepoints (pre‐ and post‐treatment), two different linear regression models were performed in R as outlined in detail in Supplement Methods Section 1.3.

### 2.6 Luciferase Reporter Assay

To investigate the functional role of the identified DMPs, the regions containing cg22187251 in *GCNT2* and cg22336863 upstream of the *ABRA* promoter were cloned into the pCpGfree basic vector (Invivogen, Belgium) to test for promoter activity and into the pCpGfree promoter vector (Invivogen, Belgium) containing a minimal promoter to test for enhancer activity. To determine the effect of DNA methylation on the activity of the region, the constructs were in vitro methylated using M.SssI (NEB, Austria). Further details are given in Supplement Methods Section 1.4. Furthermore, we obtained gene expression and DNA methylation data of *GCNT2* from lung tissue from *The Cancer Genome Atlas* (TCGA, [21]) to assess the correlation of methylation and expression in this region.

### 2.7 Analyzing Contact Points in HiC Datasets

Genome organization and chromatin interaction data (HiC) were obtained from lung tissue and visualized with 3D Genome Browser [22]. To detect potential interactions with surrounding genes, cg22187251 in *GCNT2* (chr.6: 10,529,702, hg38) was used as a reference point for virtual 4C analysis in the 3D Genome Browser.

### 2.8 GCNT2 Elisa

To assess if differential methylation of *GCNT2* results in an altered level of GCNT2 protein in whole blood samples, the GCNT2 ELISA Kit (Catalog No. ABIN6230230, Antibodies Online, USA) was performed according to the manufacturer's instructions. Since, according to the Human Protein Atlas (proteinatlas.org), GCNT2 is detectable only in amounts of 0.580 ng/mL in plasma [23] and the minimum detection limit of the ELISA is 0.313 ng/mL, undiluted whole blood samples were used.

### 2.9 Correlation of Top 100 DMP With Serological Clinical Biomarkers for AIT Responsiveness

In an exploratory approach, using a two‐tailed Spearman correlation (95% confidence interval) associations between each delta value (post‐ minus pre‐treatment) of approved serum biomarkers for AIT responsiveness (Table 1) as a proxy for the treatment effect, and the Δβ of the top 100 DMP (filtered by absolute β > 0.045; *p*‐values were non‐adjusted) per BPE‐SCIT patient (*n* = 16) were assessed. The serological AIT biomarkers include Bet v 1 and birch pollen extract (BPE) specific IgG_1_, IgG_4_, IgG, as well as the corresponding IgG_4_/IgG_1_ ratios, and biomarkers for serum inhibitory activity (inhibition ELISA, inhibition mediator release assay, facilitated antigen binding inhibition, and competition assays) and were described in detail in Aglas et al. [15]. The Spearman correlation was chosen since the biomarkers were not normally distributed. The Δβ and Δbiomarker values were used to compensate for inter‐patient variability. Only significant correlations (*p <* 0.05) were considered for further analysis. Spearman correlations were computed using GraphPad Prism 10, and significant correlations were visualized by constructing a correlation network using R (v4.5.0, igraph package [24]).

### 2.10 Statistical Analysis

*Illumina Infinium MethylationEPICv1 arrays*: *p*‐values were calculated with a paired Student's t‐test as employed in the *limma* package [19] used in the RnBeads 2.0 pipeline and further corrected for multiple testing with FDR correction of all analyzed sites.

*Cell type composition*: To calculate *p‐*values for cell type composition, Kruskal–Wallis ANOVA was applied for detecting significant changes in cell types in groups. This test was used due to unequal variances across samples and groups. To detect differences in individual cell types, a Dunn's post hoc pairwise comparison was applied.

*Luciferase reporter assays*: Welch's two sample t‐tests were performed to calculate significant differences in luciferase light signals using *p* < 0.05 as a threshold.

*Biomarker correlation analysis*: For DMPs correlating significantly with 4–6 clinical biomarkers, statistical significance was assessed using a paired t‐test for intra‐group and an unpaired t‐test for inter‐group comparison.

*Model assumptions for linear regression models*: We assessed (i) linearity with the reset test, (ii) the Breusch–Pagan test for heteroscedasticity of residuals and residual‐versus‐fitted plots, and (iii) normality of residuals was verified using Q‐Q plots and the Shapiro–Wilk test. The summary statistics are given in the Supplement Methods Section 1.3.

*Power analysis*: To determine the number of sample pairs required to detect mean beta value differences of 0.05, 0.1, or 0.2 with 80% statistical power at a *p*‐value threshold of < 1e‐6, the R package pwr. *t*‐test [25] based on a paired t‐test was used.

Unless otherwise specified, all statistical analyses were performed using R version 4.4.1 [17] and R Studio [18].

## 3 Results

### 3.1 DNA Methylation Patterns After 6 Months of Birch Pollen AIT

To analyze the effect of AIT on global DNA methylation, 16 BPE‐SCIT and 15 placebo sample pairs obtained pre‐ and post‐treatment were analyzed with the Illumina Infinium MethylationEPICv1 (850 k) array (Figure 1A). After normalization, removal of CpG positions on sex chromosomes, probes containing SNPs, and cross‐reactive probes, 690,248 total CpG probes were analyzed. Unsupervised hierarchical clustering based on Manhattan Distance showed clustering of sample pairs (pre‐ and post‐treatment of each sample clustered in one branch, Figure 1B; Figure S1). No distinction between BPE‐SCIT‐ and placebo‐treated patients was observed at the global DNA methylation level. Technical replicates of sample A4 (A4_post_1 and A4_post_2, Figure 1B) as well as placebo sample P1 (P1_post_1 and P1_post_2) clustered in one branch confirming the reproducibility of our analysis. PCA of Δβ of post‐ versus pre‐treatment of the 100 top DMPs exposed clustering of BPE‐SCIT patients mainly across Q3 (Figure S2). The difference in distribution on the top 100 DMPs by patient between Q3, containing 10 BPE‐SCIT patients, and Q2 (7 placebo and 1 BPE‐SCIT patient) was significant (*p* < 0.0001), indicating that AIT induces DNA methylation changes. Since the Kaiser–Meyer–Olkin (KMO) measure and Bartlett's test of sphericity indicated that the data were not suitable for PCA, we opted for uniform manifold approximation and projection (UMAP) instead. UMAP analysis showed a significant sample clustering by treatment based on a permanova test (*p* = 0.031, Figure 1C; Supplement Methods Section 1.2; Table S1).

Differential methylation was calculated as intra‐pair difference between each sample for pre‐ and post‐treatment. In total, 20 FDR significant DMPs were detected for BPE‐SCIT post‐ versus pre‐treatment (Table S2), of which one showed a difference in |Δβ| > 0.045 (Table 2). This significantly hypermethylated position cg22187251 was identified in exon 2 of glucosaminyl (N‐acetyl) transferase 2 (*GCNT2*, Figure 1D,E). Additionally, a second hypermethylated CpG cg22336863 with β > 0.045 was found upstream of the transcription start site of actin‐binding Rho‐activating protein (*ABRA*), with an FDR‐adjusted *p*‐value of 0.068, indicating a borderline significance that might reach statistical significance with a larger sample size (Table 2; Table S2; Figure S3). Moreover, a differentially methylated region overlapping the HLA gene complex on chromosome 6 (chr6: 28,749,781–37,012,716) was detected. Although five hypomethylated DMPs reached statistical significance in this region, the |Δβ| was rather minor (< 0.045) and therefore not informative (Figure S4; Table S3). No significant changes were obtained for the placebo‐treated group (Figure S5; Table S4).

**TABLE 2:** Top differentially methylated positions (DMPs) for post‐ compared to pre‐BPE‐SCIT treatment.

No.	CG ID	Chr	Position	CGI relation	Gene	Δβ	unadj. *p*	FDR adj. *p*	Gene
1	cg22187251	chr6	10,529,702	Open Sea	*GCNT2*	0.048	4.88E‐09	0.00328	*GCNT2*
2	cg22336863	chr8	106,770,848	Open Sea	*ABRA*	0.068	2.32E‐06	0.06775	*ABRA*

*Note:* Pairwise sample comparison between post‐ versus pre‐BPE‐SCIT treatment in 16 sample pairs resulted in 2 significant differentially methylated positions with a mean difference in DNA methylation |Δβ| > 0.045. Each row represents a single CpG site identified by its Illumina probe ID (CG ID), chromosomal location (Chr, Position) given in assembly hg38, and relation to a CpG island (CGI Relation; e.g., island, shore, shelf, open sea). The DNA methylation difference between groups is given as mean difference in beta value (Δβ). Significance is shown by the unadjusted *p*‐value (unadj. *p*) and the false discovery rate‐adjusted *p*‐value (FDR adj. *p*). Associated genes (Gene) are annotated based on proximity to the CpG site. Sites with FDR adj. *p* < 0.05 were considered statistically significant.

Since blood is composed of a heterogeneous mix of cell types, changes in cell type composition can lead to differences in DNA methylation that may be unrelated to the disease or the treatment itself. To account for this, cell type composition was analyzed. After BPE‐SCIT treatment, a significant increase in B‐ (1.9‐fold difference) and CD8+ T‐cell (2.0‐fold difference) proportions and a reduction in NK‐cells (0.03‐fold difference) was detected (Table 3; Figure 1F upper panel). In addition, placebo‐treated patients also showed an increased B‐cell proportion, to the same extent as samples from the BPE‐SCIT‐treated patients (Figure 1F lower panel). However, proportions of CD8+ T‐cells and NK‐cells remained unchanged, indicating a BPE‐SCIT‐specific treatment effect.

**TABLE 3:** Effect of birch pollen extract‐based subcutaneous allergen immunotherapy (BPE‐SCIT) and placebo treatment on cell type proportions.

Treatment	Cell type	Mean proportion	SE	Mean proportion	SE	BH adj. *p*
		Pre‐treatment		Post‐treatment		
BPE‐SCIT	**B‐cell**	**0.030**	**0.005**	**0.056**	**0.008**	**0.0127**
	CD4 T	0.224	0.026	0.196	0.017	0.372
	**CD8 T**	**0.069**	**0.027**	**0.140**	**0.021**	**0.046**
	Mono	0.094	0.005	0.089	0.005	0.442
	Neu	0.529	0.019	0.547	0.016	0.473
	**NK**	**0.037**	**0.011**	**0.001**	**0.010**	**0.020**
Placebo	**B‐cell**	**0.031**	**0.006**	**0.055**	**0.008**	**0.030**
	CD4 T	0.242	0.025	0.236	0.014	0.832
	CD8 T	0.031	0.016	0.057	0.022	0.336
	Mono	0.076	0.005	0.078	0.005	0.759
	Neu	0.560	0.026	0.579	0.021	0.578
	NK	0.034	0.011	0.012	0.013	0.193

*Note:* Mean values and standard error of the mean (SE) of estimated cell type proportions are shown. Significant changes between pre‐ and post‐treatment are marked in bold. *p*‐values were calculated with pairwise comparisons using a t‐test with Benjamini Hochberg (BH adj. *p*) correction.

The analyses of cg22187251 revealed that this probe varied significantly among cell types, with CD4+ and CD8+ T cells and natural killer cells showing the highest methylation with β at approximately 0.6 and the remaining cell types at 0.15, which was not the case for cg22336863 (Figure S6). According to the cell type analysis, BPE‐SCIT‐treated patients had increased ratios for B‐ and CD8+ T cells but decreased values for NK cells (Figure 1F upper panel). These values account for < 1% increased mean methylation post‐ vs. pre‐treatment in the BPE‐SCIT group and less than 0.5% methylation increase in the placebo group. When performing a linear regression model including all cell types as well as the timepoint of sample drawing, the strongest, but non‐significant, correlations were treatment for the BPE‐SCIT group, pre‐ vs. post‐treatment (*p* = 0.069), and B‐cell proportion for the placebo group (*p* = 0.0716, Supplement Methods Section 1.3). On this basis, we can exclude that the observed hypermethylation is solely caused by cell type variation.

### 3.2 *GCNT2* cg22187251 Harbors Promoter and Enhancer Regulatory Functions

**FIGURE 2:**
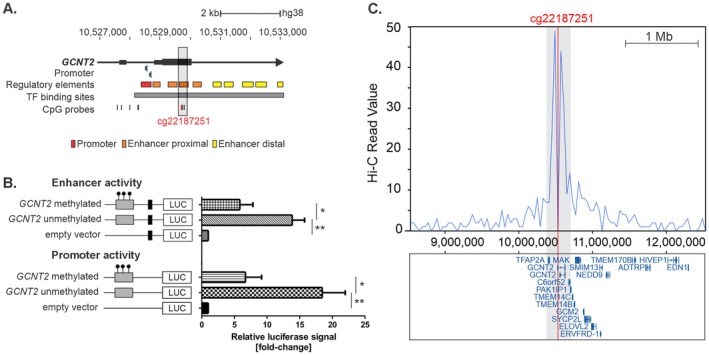
cg22187251 harbors regulatory functions. (A) Shows the genomic annotation, promoters, regulatory elements and transcription factor binding regions in the *GCNT2* gene overlapping the differentially methylated site cg22187251. Furthermore, CpG probe locations from the Infinium MethylationEPIC array are depicted. The gray highlighted region was analyzed in a luciferase reporter assay. (B) Luciferase reporter assays using CpGfree vectors revealed promoter and enhancer functions of the region containing cg22187251, which was reduced when the insert was methylated. Pairwise comparisons against the empty vector with Welch two sample *t*‐test (C) HiC data analysis showed that cg22187251 contacts several up‐ and downstream regions, but most significantly the *GCNT2* and the *C6orf52* gene body (the blue peaks with highest HiC signals); *p < 0.05, **p < 0.01.

cg22187251 is located in exon 3 of *GCNT2* within an enhancer proximal region harboring several binding sites for different transcription factors (Figure 2A). To test for a putative methylation‐dependent promoter or enhancer function, luciferase reporter assays were performed. An 18‐fold (±3.5 SE, *p* = 0.004, Figure 2B) promoter function and a 14‐fold (±1.9 SE, *p* = 0.001) enhancer function was detected in the case of an unmethylated *GCNT2* insert. Methylation reduced the promoter function to 7‐fold (±2.5, SE, *p* = 0.09) and the enhancer function to 6‐fold (±2.0 SE, *p* = 0.08) compared to the empty vector, pointing clearly towards a methylation dependent regulatory function of the detected region. This aligns with the observed correlation between DNA methylation and gene expression in lung tissue, where higher methylation levels of cg22187251 were associated with lower expression of *GCNT2* in B‐cells, lung, liver and breast tissue as reported in *The Cancer Genome Atlas* (TCGA, [21]; Figure S7).

Analysis of published HiC data in the 3D genome browser showed that cg22187251 contacts an upstream region of *GCNT2* as well as its downstream gene body and C6orf52 (protein coding gene), underlining the detected enhancer function from the luciferase reporter assay (Figure 2C).

A GCNT2‐specific sandwich ELISA was performed to investigate if changes in DNA methylation are also reflected on the protein level in the whole blood samples (Figure S8). All observed OD values were below the detection limit of the assay, most likely due to the low concentration of GCNT2 in blood.

Similarly, the reporter assay demonstrated that the identified DMP cg22336863, located upstream of the *ABRA* promoter (Figure S9A), exhibited significant enhancer activity in a methylation‐dependent manner, while no promoter activity was observed (Figure S9B).

### 3.3 Spearman Correlations With Clinical Biomarkers

**FIGURE 3:**
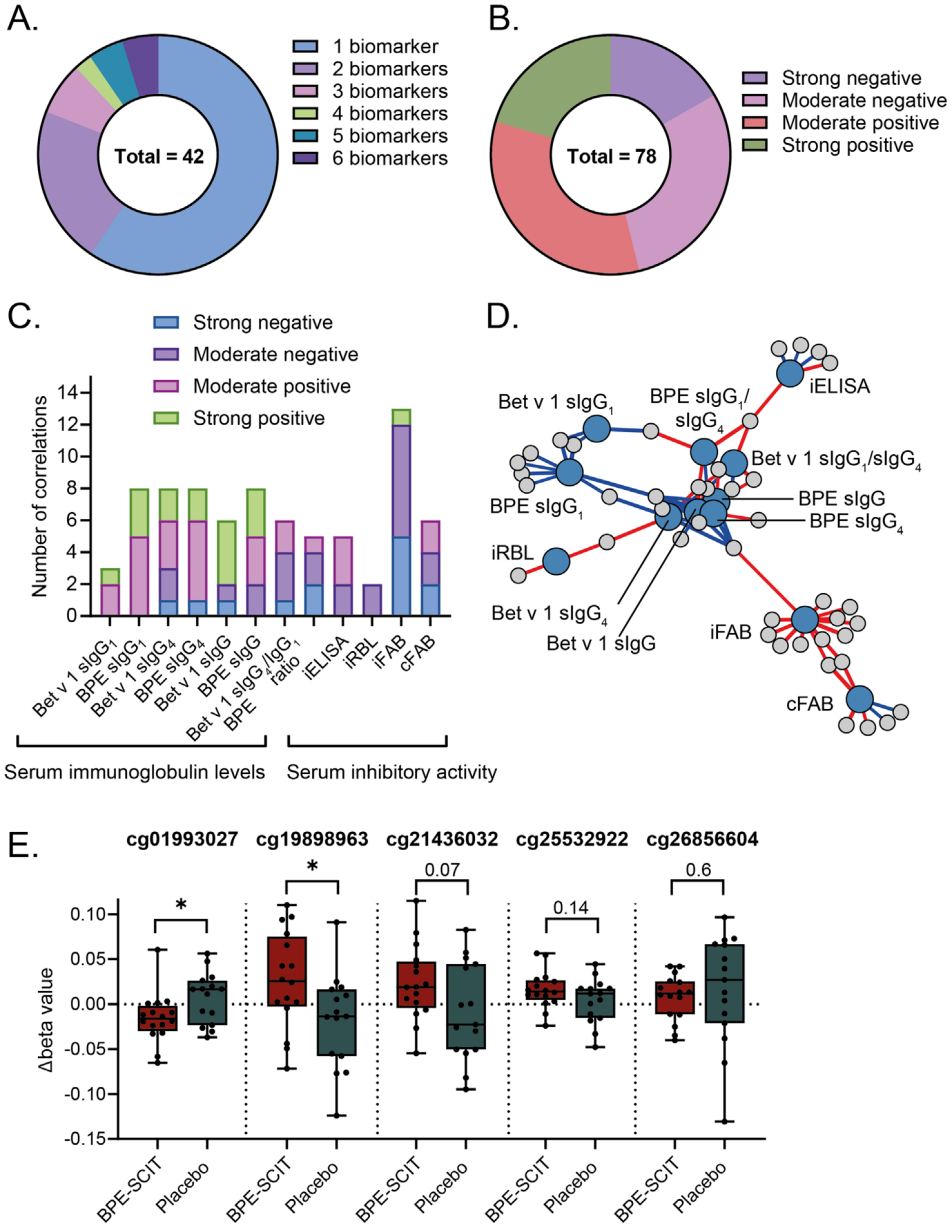
Spearman correlation of 100 top differentially methylated probes (DMPs, filtered by |Δβ| > 0.045) with serum biomarkers as proxy for AIT efficacy. (A) In total, 42 of the top 100 DMPs correlated significantly with at least 1 biomarker. Of those, 25 (59.52%) correlated with 1 biomarker, 9 (21.43%) with 2, 3 (7.14%) with 3, 1 (2.38%) with 4, 2 (4.76%) with 5, and 2 (4.76%) with 6 biomarkers. (B) Overall, 78 significant correlations were identified, of which 13 showed a strong negative correlation, 23 a moderate negative correlation, 26 a moderate positive correlation and 16 a strong positive correlation. Moderate correlations were defined as −0.60 to −0.40 and 0.40 to 0.60, and strong correlations as −0.80 to −0.60 and 0.60 to 0.80. (C) The number of correlations distributed across the various biomarker types, with positive correlations mainly observed for the various immunoglobin G levels, while negative correlations were mainly observed for our read‐outs for serum inhibitory activity (2 for iELISA, 2 iRBL, 13 iFAB and 4 cFAB). (D) Spearman correlation network with nodes representing biomarkers (blue) and DMPs (gray), and edge color direction of correlation (blue = positive, red = negative). (E) DNA methylation (Δβ values) of cg01993027 (*SRSF4*), cg19898963 (*LRRC7*), cg21436032, cg26856604 (*KIF1B*) and cg25532922 (*LBH*), correlating significantly with 4–6 clinical biomarkers. Statistical significance was assessed using an unpaired *t*‐test for inter‐group comparison. *p < 0.05. cFAB, facilitated antigen binding competition assay; IELISA, inhibition ELISA; iFAB, facilitated antigen binding inhibition assay; iRBL, inhibition mediator release assay.

Since neither one of the two identified DMPs (cg22187251‐*GCNT2* and cg22336863‐*ABRA*) correlated with the clinical biomarkers associated with the treatment response (Table 1), we performed an exploratory in addition to the conservative approach using the top 100 DMPs (BPE‐SCIT post‐ vs. pre‐treatment) for analysis of direct correlations with the biomarkers representative for the treatment effect. Out of those DMPs 42% significantly correlated with at least one of the clinical biomarkers, of which five DMPs (cg01993027‐*SRSF4*, cg19898963‐*LRRC7*, cg21436032, cg26856604‐*KIF1B* and cg25532922‐*LBH*) even significantly correlated with ≥ 4 clinical biomarkers (Figure 3A–D). The absolute Spearman r ranged between 0.52 and 0.75, indicating a moderate to strong correlation. The five DMPs mainly correlated with Bet v 1 and BPE specific IgG_4_, IgG, as well as the IgG_4_/IgG_1_ ratios (Figure S10). Comparing the Δβ values revealed significant differences in BPE‐SCIT vs. placebo for cg01993027 and g19898963 with a mean difference of 2.28% and 4.49%, respectively, and a trend for cg21436032 with 3.21% mean difference in methylation. Of note, cg01993027 was hypomethylated while the other two were hypermethylated in BPE‐SCIT vs. placebo controls (Figure 3E; Figure S11).

## 4 Discussion

To the best of our knowledge, this study represents the first EWAS investigating DNA methylation changes in individuals before and after treatment with BPE‐SCIT or placebo in birch pollen allergy directly in patients' blood, without any culturing or restimulation of cells prior to analysis as reported here [26]. Our findings revealed in total 20 DMPs with an FDR *p* < 0.05, of which one showed a mean |Δβ| > 0.045 (cg22187251, *GCNT2*). Patients treated with BPE‐SCIT showed hypermethylation of cg22187251 after treatment compared to pre‐treatment levels, whereas no effect was observed in the placebo group. DNA methylation data for cg22187251 from healthy individuals from the large European Mechanisms of the Development of Allergy (MeDALL) study [10, 27] showed that allergic patients have lower methylation levels compared to healthy controls, although the difference was statistically not significant (correspondence with Prof. Dr. Koppelman; β = −0.0051, SE = 0.0050, *p* = 0.314, [27]). Finally, albeit only barely significant, cg22336863 (*ABRA*) showed hypermethylation, along with hypomethylation of several DMPs within the HLA gene complex. This suggests that antigen processing may be modulated by birch pollen AIT [28].

Successful AIT is usually characterized by an early—occurring within the first months of AIT—suppression of IgE‐mediated activation of mast cells and basophils, followed by an induction of regulatory T and B cells, accompanied by a production of IgG antibodies competing with IgE for allergen recognition; altogether shaping the patient's immunotolerance to allergen exposure [29, 30]. Our DNA methylation‐based cell type proportion analysis revealed that birch pollen AIT decreased the proportions of NK and increased the CD8+ T cell population, while the increase in B cells was also observed for placebo‐treated patients, which is likely explained by the adjuvant aluminum hydroxide known to induce B cell proliferation independently of a specific antigen [31, 32]. At first glance, our findings that AIT induced an increase in CD8+ T cells on the methylome level are rather surprising since CD8+ T cells are rather known for their role in viral infection. This is further interesting because during viral infections the proliferation of CD8+ is usually accompanied by an expansion of NK cells, which was not the case in our study where the NK cell frequency dropped. In respect of AIT, CD8+ T cells were found to take over important regulatory functions in the induction of immunotolerance [33, 34, 35, 36]. Only recently, Tsai et al. described an immunosuppressive function of CD8+ CD25+ CD137+ Treg induced by house dust mite SCIT. Stimulation of the CD8+ CD25+ CD137+ Tregs resulted in secretion of IL‐10 and TGF‐β [34]. CD8+ T cells were also studied in a murine allergy model and observed to dampen mucus secretion and the influx of eosinophils in sensitized mice [35]. Since our results are based on methylome data, our findings have to be validated on the cellular level; for example, by flow cytometry or single‐cell RNA sequencing, in future studies.

The glucosaminyl (N‐acetyl) transferase 2 gene (*GCNT2*) is expressed on several immune cells including basophils, dendritic cells, and naïve B cells [23]. In a mouse model of food antigen‐induced anaphylaxis, it has been shown that *GCNT2* was upregulated in dendritic cells [37]. Furthermore, *GCNT2* was hypermethylated in a study investigating DNA methylation of 11‐year‐old patients with respiratory allergy compared to healthy controls, albeit it was not indicated which CpG positions were affected [38]. This clearly highlights that *GCNT2* plays a role in allergic diseases.

Several studies have investigated the mechanistic function of *GCNT2* in different immune cells, which could explain the impact on allergic diseases. In naïve B cells, the expression of *GCNT2* was downregulated to retain enough of the immunoregulatory lectin Galectin 9 (Gal‐9) to bind CD45 and to attenuate B cell activation, regulate B cell receptor (BCR) signaling, and to influence B cell activation, suggesting differential regulation of BCR signaling by Gal‐9 [39]. Besides, retained Gal‐9 binds IgE bound to the mast cell Fc epsilon receptor (FcεR) and prevents degranulation induced by allergen‐IgE cross‐linking [40, 41]. Zhang et al. demonstrated that downregulated expression of *GCNT2* increases epithelial marker E‐cadherin levels [42] and, at the protein level, that GCNT2 directly influences the canonical TGF‐β pathway (SMAD‐dependent), suggesting an involvement of GCNT2 in mediating immunotolerance [42]. E‐cadherin negatively regulates cell proliferation and migration by inhibiting Ras homolog family member A (RhoA) [43]. In dendritic cells, activated RhoA signaling influences cytoskeleton rearrangement, which regulates their migration and T cell's stimulatory capacity [44]. RhoA‐deficient mice showed a significant reduction in CD4+ T cells and concomitant inhibition of Th2‐differentiation potential, as well as attenuated allergic airway inflammation, pointing out the critical role of RhoA in asthma development [45]. Of note, in our data, the second site which was hypermethylated in the BPE‐SCIT‐treated group was Actin‐binding and Rho activating protein (*ABRA*).

In line with the modulation of immunotolerance by GCNT2, is our finding that cg01993027‐*SRSF4*, which correlated with Bet v 1 and BPE sIgG_4_, sIgG, and the sIgG_4_/sIgG_1_ ratio, was hypomethylated in comparison to placebo. The serine/arginine‐rich splicing factor 4 (*SRSF4*) is overexpressed in the nasal epithelium and PBMCs; however, its specific role, other than the involvement in alternative splicing for the expression of Tau isoforms and modulation of neuroimmune responses, is unclear [46]. Other splicing factors of the same family, such as *SRSF1*, were shown to be essential for Treg homeostasis and activity, as observed in systemic lupus erythematosus patients [47], but also for regulation of inflammatory responses in macrophages and of antiviral responses [48, 49]. For cg19898963‐*LRRC7*, which also correlated with the same six biomarkers and was significantly hypermethylated compared to placebo, no obvious association with immunoregulation has been reported. Even though, in a patient with B cell lymphoma (i.e., a cancer type resulting in impaired antibody production of B cells), a single nucleotide variation in *LRRC7* was identified, which might indicate a role of this gene in regulation of antibody production in B cells [50].

A key strength of this pilot study lies in its longitudinal, paired‐sample design, which enables robust detection of within‐subject DNA methylation changes pre‐ and post‐treatment. While larger EWAS studies comparing allergic and healthy individuals often do not apply stringent beta value thresholds (e.g., [8, 27]), we deliberately applied a conservative beta threshold in light of our limited sample size. This rigorous approach strengthens the reliability of our findings and supports that top hits such as *GCNT2* are likely to represent true positive signals. The limitation of our study is that a rather small cohort was used (*n* = 15–16 patients per group). For genome‐wide DNA methylation studies, especially if the effect size is considered to be low or modest, larger cohort sizes improve the resolution of findings and statistical power. However, even with a paired design to detect minor changes in DNA methylation of 5%, we have calculated that 538 samples (150 sample pairs for a 10% difference) would be required to reach a power of 80% with *p* < 1e‐6 (Figure S12), which is not feasible for small exploratory clinical studies. Also, by using whole blood samples for DNA extraction instead of PBMCs, where there is no influence of granulocytes comprising ~50% of whole blood composition, resolution is lowered. Together, these factors are likely the reason for low Δβ and only a few significant observations in our study. In the future, replicating our preliminary data using an alternative approach (e.g., Oxford Nanopore Technology) is of utmost importance.

Wang et al., the only other EWAS study investigating AIT in allergic patients, in this case house dust mite allergy, performed the analysis of a 450 k array on allergen‐restimulated PBMCs [26]. In contrast, in our study the cells were not restimulated, which would result in proliferation and enrichment of allergen‐specific cell types such as T and B cells. These data must be interpreted with caution, since stimulation and proliferation of cells induce changes in the DNA methylation landscape [51, 52], and therefore probably do not entirely reflect the situation in the patient. The study identified three DMPs (cg21272897, cg02017208, and cg18146226) significantly altered in AIT patients compared to allergic asthma controls. While cg21272897 is no longer covered on the 850K array, cg02017208 and cg18146226 were not represented in our dataset, since we filtered out SNP probes to avoid allele‐specific methylation artifacts and misclassification of methylation changes due to CpG site alterations.

## 5 Conclusion

In conclusion, BPE‐SCIT treatment caused hypermethylation of two genes *GCNT2* and *ABRA*, which are associated with RhoA signaling, modulation of the Th2 response, and immunotolerance induction via the TGF‐β pathway. Together, these events are involved in modulating the allergic immune response by AIT. The hypermethylated *GCNT2* cg22187251 and *ABRA* cg22336863 are located in methylation‐dependent regulatory region, and our luciferase reporter assays have shown that increased methylation caused a significant reduction in promoter and enhancer functions.

Despite the limited sample size, our first pilot study provides evidence for DMPs associated with BPE‐SCIT treatment. Further research with larger cohorts and extended treatment durations will enhance the detection of significant DMPs and regions and deepen our understanding of epigenetic regulation in AIT.

## Author Contributions

A.L. conducted the genome‐wide DNA methylation analysis. A.L., V.E., and M.H. performed luciferase reporter experiments. A.L., M.H., and L.A. wrote the manuscript and created the figures. R.R. designed, organized, and coordinated the clinical trial. All authors read the manuscript and contributed to the discussions and revisions of the manuscript.

## Ethics Statement

The study (ClinicalTrials.gov Identifier: NCT04912076) was approved by the Danish Medicines Agency and The Regional Committees on Health Research Ethics for Southern Denmark (EudraCT number 2018‐001486‐17).

## Conflicts of Interest

Ronald van Ree received consulting fees and/or speaker fees from Angany Inc., HAL Allergy BV, Citeq BV, ThermoFisher Scientific, ALK Abello, Reacta Healthcare Ltd., Mission MightyMe, and The Protein Brewery and has stock options from Angany Inc. The remaining authors declare no conflicts of interest.

## Supporting information


Appendix S1.

## Data Availability

According to the obtained informed consent from each patient, data cannot be made publicly accessible.
