# Supplementary material for: Genome‐Wide Blood DNA Methylation Profiling in Birch Pollen Allergic Patients Undergoing Allergen‐Specific Immunotherapy
Source: Allergy. 2025 Oct 9;80(12):3412–23. doi: 10.1111/all.70094 (PMC12666740; doi:10.1111/all.70094)
Supplement: Supplementary file 1 — Appendix S1. [file ALL-80-3412-s001.docx]

**SUPPLEMENT**

**Genome-wide blood DNA methylation profiling in birch pollen allergic patients undergoing allergen-specific immunotherapy**

Angelika Lahnsteiner^1,2^, Victoria Ellmer^1,2^, Mengzhen Hao^1,2^, Line Kring Tannert^3^, Serge A. Versteeg^4^, Carsten Bindslev-Jensen^3,5^, Ronald van Ree^4,6^, Angela Risch^1,2^, Lorenz Aglas^1,7^

Affiliations:

^1^Department of Biosciences and Medical Biology, Paris Lodron University of Salzburg, Austria

^2^Center for Tumor Biology and Immunology (CTBI), Paris Lodron University of Salzburg, Austria
^3^Odense Research Center for Anaphylaxis, Odense University Hospital, Odense, Denmark

^4^Department of Experimental Immunology, Amsterdam University Medical Centers, location AMC, Amsterdam, The Netherlands

^5^Department of Dermatology and Allergy Center, Odense University Hospital, Odense, Denmark

^6^Department of Otorhinolaryngology, Amsterdam University Medical Centers, location AMC, Amsterdam, The Netherlands

^7^Institute of Pathophysiology and Allergy Research, Center for Pathophysiology, Infectiology and Immunology, Medical University of Vienna, Vienna, Austria.

Table of Contents

[1. Supplement Methods 3](#_Toc200697341)

[1.1. Differential Methylation analysis 3](#_Toc200697342)

[1.2. Dimensionality reduction 4](#_Toc200697343)

[1.3. Linear regression model 5](#_Toc200697344)

[1.4. Luciferase reporter assay 9](#_Toc200697357)

[2. Supplement Tables 11](#_Toc200697358)

[3. Supplement Figures 17](#_Toc200697359)

[4. References 25](#_Toc200697360)

# **Supplement Methods**

## **Differential Methylation analysis**

DNA methylation analysis was performed according to the pipeline below. after importing the raw files in IDAT format and subsequent quality control, probes without values, or low coverage and CpGs on sex chromosomes were excluded. Data normalization was performed via beta-mixture quantile normalization (BMIQ, (1)). Surrogate variable analysis (SVA) was performed to correct for unknown cofounders and batch or chip effects (2). To account for cell type heterogeneity, we applied the Houseman et al. algorithm (3) by using a reference methylome dataset based on the DNA methylation profiles of purified blood cell populations (4). Cell type proportions were estimated on the top 50,000 CpGs with the highest variance across all samples (5).

Differentially methylated positions (DMPs) were called by using a paired comparison of each sample pair (pre- and post-treatment) using the empirical Bayes method ‘limma’ (6) and identified as statistically significant with a false discovery rate- (FDR-) corrected *p*<0.05. To avoid the detection of false positives, we additionally applied an absolute mean beta value difference (|Δβ|) threshold of |Δβ|>0.045. In addition, two technical replicates were included, one for BPE-SCIT sample A4 and one for placebo sample P1, to evaluate reproducibility. The normalization methods are explained in detail in the sections below:

- - 1. **Beta-mixture quantile (BMIQ) normalization method of raw methylation data**

Illumina Infinium DNA methylation arrays use two distinct probe types (Type I and Type II), which differ in chemistry and performance, introducing systematic bias in β-values that can confound downstream analyses. Especially type II probes have been shown to be biased and less reproduceable (7). The assumption-free beta-mixture quantile (BMIQ) normalization method corrects this bias by aligning the distributions of Type II probes to those of Type I probes, which are considered more reliable. BMIQ works by fitting a three-component beta mixture model to Type I probe data, representing unmethylated, partially methylated, and fully methylated states. It then assigns Type II probes to these methylation states and applies quantile normalization within each class to match the corresponding Type I distribution. Details on the applied model can be found in very detail in the original publication (1).

- - 1. **Surrogate Variable Analysis (SVA)**

High-dimensional biological data such as DNA methylation or gene expression profiles are often influenced by hidden sources of variation, including batch or chip effects, technical noise, or unknown biological factors like cell passage numbers or age. Surrogate Variable Analysis (SVA) is a statistical framework that estimates these unmeasured confounders and incorporates them into the analysis to improve detection of true biological signals (2). These surrogate variables are then included as covariates in downstream linear modeling in ‘limma’ (6) as implemented in the RnBeads 2.0 pipeline (5).

- - 1. **Cell-type composition detection with Houseman method**

The Houseman method is a reference-based algorithm used to estimate the proportions of different blood cell types from DNA methylation data. It assumes that the methylation level at selected CpG sites in a mixed (bulk) sample is a linear combination of methylation levels from pure cell types. Using a reference methylation dataset from sorted blood cell types, the method (i) selects informative CpG sites which are by default the 50,000 CpGs with the highest variance across all samples, (ii) applies constrained linear regression to estimate how much each cell type contributes to the overall methylation signal and (iii) outputs estimated cell type proportions (as indicated in table 3), which are then used as covariates to adjust for variations in the cell-type composition. Further details can be found in the original publication (3).

## **Dimensionality reduction**

Uniform Manifold Approximation and Projection (UMAP) is a dimensionality reduction technique designed to uncover the underlying structure of complex datasets (8). By integrating principles from manifold learning and topological data analysis, UMAP enables the visualization of high-dimensional data in lower-dimensional spaces. Unlike methods such as PCA or t-SNE, UMAP excels at preserving global data structure while maintaining computational efficiency, making it especially suitable for visualizing large datasets and identifying patterns that might be missed by other approaches. As such, UMAP offers a powerful tool due to its ability to capture non-linear relationships and complexities in the analyzed datasets. Since DNA methylation data often exhibit nonlinear patterns due to high variability across genomic regions and their complex relationship with gene expression, UMAP outperforms linear methods such as principal component analysis (PCA) (9).

UMAP was calculated using the Manhattan distance, 15 neighbors, and 3 components, based on the differences in beta values between post- and pre-treatment for the top 100 differentially methylated positions (DMPs), sorted by unadjusted p-value. The number of neighbors was set to 15 to preserve local structures, as the DNA methylation changes represented by differences in beta values were relatively small. To test for significant clustering, we applied a permutation test for adonis under reduced model using the adonis2 R-package with Manhattan distance (10). The table below shows the permutational multivariate analysis of variance (PERMANOVA, adonis) for the reduced model. Df represents the degrees of freedom for each term, SumOfSqs is the sums of squared distances attributed to the term, R² describes the proportion of total variance explained (effect size), F is the pseudo‑F statistic comparing term‐associated spread to residual spread, and Pr(>F) represents the permutation *p*‑value. Asterisks denote significant terms (*p*< 0.05).

|  | **Df** | **SumOfSqs** | **R2** | **F** | **Pr(>F)** |
| --- | --- | --- | --- | --- | --- |
| Model | 1 | 18.1361425 | 0.12771263 | 4.245924451 | **0.031 *** |
| Residual | 29 | 123.8712884 | 0.87228737 |  |  |
| Total | 30 | 142.0074309 | 1 |  |  |

## **Linear regression model**

## **Summary statistics**

Since linear regression models were used to assess cell-type specific changes in differential expression of healthy versus patients (11)., we utilized this method to test for a dependency of DNA methylation at cg221827251. Therefore, two different linear regression models were applied to the datasets:

$$\boldsymbol{model}\boldsymbol{1} <- lm\left( Methylation \sim Neu + NK + Bcell + CD4T + CD8T + Mono + Timepoint, data = df \right)$$

$$\boldsymbol{model}\boldsymbol{2}<- lm(Methylation \sim Neu * Timepoint + NK * Timepoint +$$

$$Bcell * Timepoint + CD4T * Timepoint + CD8T * Timepoint + Mono * Timepoint, data = df)$$

Below the summary statistics is given for the BPE-SCIT-treated and the placebo-treated patients.

## **BPE-SCIT treatment:**

Call:

lm(formula = Methylation ~ Neu + NK + Bcell + CD4T + CD8T + Mono +

Timepoint, data = df)

Residuals:

Min 1Q Median 3Q Max

-0.10070 -0.03320 0.00911 0.03610 0.11497

Coefficients:

| **Term** | **Estimate** | **Std. Error** | **t value** | **Pr(>\|t\|)** |
| --- | --- | --- | --- | --- |
| (Intercept) | -0.06014 | 0.73720 | -0.082 | 0.9362 |
| Neu | 0.47620 | 0.79347 | 0.600 | 0.5587 |
| NK | -0.05937 | 1.29615 | -0.046 | 0.9642 |
| Bcell | -0.37116 | 0.98736 | -0.376 | 0.7130 |
| CD4T | 0.33239 | 0.59367 | 0.560 | 0.5851 |
| CD8T | 0.62714 | 0.65687 | 0.955 | 0.3571 |
| Mono | -0.37764 | 1.14759 | -0.329 | 0.7473 |
| Timepoint_2 vs Timepoint_1 | -0.06551 | 0.03308 | -1.980 | **0.0692** |

lm(formula = Methylation~ Neu * Timepoint + NK * Timepoint + Bcell *

Timepoint + CD4T * Timepoint + CD8T * Timepoint + Mono *

Timepoint, data = df)

Coefficients:

| **Term** | **Estimate** | **Std. Error** | **t value** | **Pr(>\|t\|)** |
| --- | --- | --- | --- | --- |
| (Intercept) | 1.3574 | 2.0943 | 0.648 | 0.538 |
| Neu | -1.1694 | 2.3855 | -0.490 | 0.639 |
| posttreatment vs pretreatment | -3.8792 | 3.9102 | -0.992 | 0.354 |
| NK | -2.7791 | 3.9267 | -0.708 | 0.502 |
| Bcell | -2.0281 | 2.0243 | -1.002 | 0.350 |
| CD4T | -0.3501 | 1.3788 | -0.254 | 0.807 |
| CD8T | -0.2291 | 1.6882 | -0.136 | 0.896 |
| Mono | -2.3065 | 2.6733 | -0.863 | 0.417 |
| Timepoint_2 vs Timepoint_1:Neu | 3.8827 | 4.0089 | 0.969 | 0.365 |
| Timepoint_2 vs Timepoint_1:NK | 7.5600 | 6.4818 | 1.166 | 0.282 |
| Timepoint_2 vs Timepoint_1:Bcell | 7.6146 | 5.8623 | 1.299 | 0.235 |
| Timepoint_2 vs Timepoint_1:CD4T | 2.8485 | 3.5232 | 0.809 | 0.445 |
| Timepoint_2 vs Timepoint_1:CD8T | 2.8801 | 3.6770 | 0.783 | 0.459 |
| Timepoint_2 vs Timepoint_1:Mono | 4.8125 | 4.8273 | 0.997 | 0.352 |

## **Placebo treatment:**

Call:

lm(formula = Methylation ~ Neu + NK + Bcell + CD4T + CD8T + Mono +

Timepoint, data = df)

Coefficients:

| **Term** | **Estimate** | **Std. Error** | **t value** | **Pr(>\|t\|)** |
| --- | --- | --- | --- | --- |
| (Intercept) | -0.26104 | 0.94618 | -0.276 | 0.7867 |
| Neu | 0.24124 | 0.97220 | 0.248 | 0.8076 |
| NK | 1.57066 | 1.63288 | 0.962 | 0.3524 |
| Bcell | 2.88794 | 1.48188 | 1.949 | **0.0716** |
| CD4T | 0.34556 | 0.95506 | 0.362 | 0.7229 |
| CD8T | 0.56105 | 0.95161 | 0.590 | 0.5649 |
| Mono | 1.05709 | 1.00767 | 1.049 | 0.3119 |
| Timepoint_2 vs Timepoint_1 | 0.02589 | 0.04147 | 0.624 | 0.5424 |

Call:

lm(formula = Methylation~ Neu * Timepoint+ NK * Timepoint+ Bcell *

Timepoint+ CD4T * Timepoint+ CD8T * Timepoint+ Mono *

Timepoint, data = df)

Coefficients:

| **Term** | **Estimate** | **Std. Error** | **t value** | **Pr(>\|t\|)** |
| --- | --- | --- | --- | --- |
| (Intercept) | -1.1189 | 1.8565 | -0.603 | 0.563 |
| Neu | 1.3172 | 1.7923 | 0.735 | 0.483 |
| posttreatment pretreatment | -2.4279 | 2.4123 | -1.006 | 0.344 |
| NK | 3.2616 | 3.5011 | 0.932 | 0.379 |
| Bcell | 1.4518 | 3.0829 | 0.471 | 0.650 |
| CD4T | 0.9637 | 2.1201 | 0.455 | 0.661 |
| CD8T | 1.8061 | 1.8991 | 0.951 | 0.369 |
| Mono | 2.0049 | 1.4579 | 1.375 | 0.206 |
| Timepoint_2 Timepoint_1:Neu | 2.3092 | 2.4065 | 0.960 | 0.365 |
| Timepoint_2 Timepoint_1:NK | 2.1435 | 4.1140 | 0.521 | 0.616 |
| Timepoint_2 Timepoint_1:Bcell | 6.5520 | 3.7922 | 1.728 | 0.122 |
| Timepoint_2 Timepoint_1:CD4T | 2.9981 | 2.6730 | 1.122 | 0.295 |
| Timepoint_2 Timepoint_1:CD8T | 2.1416 | 2.4748 | 0.865 | 0.412 |
| Timepoint_2 Timepoint_1:Mono | 1.1467 | 2.0611 | 0.556 | 0.593 |

## **Test for linearity**

## **Alutard treatment:**

| **Model1:** | **Model 2:** |
| --- | --- |
| RESET = 0.10529, df1 = 2, df2 = 22,  p-value = 0.9005 | RESET = 0.20838, df1 = 2, df2 = 16,  p-value = 0.8141 |

## **Placebo treatment:**

| **Model1:** | **Model 2:** |
| --- | --- |
| RESET = 1.6825, df1 = 2, df2 = 20,  p-value = 0.2112 | RESET = 1.8459, df1 = 2, df2 = 14,  p-value = 0.1943 |

## **Breusch-Pagnan test for heteroscedasticity**

## **Alutard treatment:**

| **Model1:** | **Model 2:** |
| --- | --- |
| studentized Breusch-Pagan test  BP = 5.0242, df = 7, p-value = 0.657 | studentized Breusch-Pagan test  BP = 14.656, df = 13, p-value = 0.3293 |
| 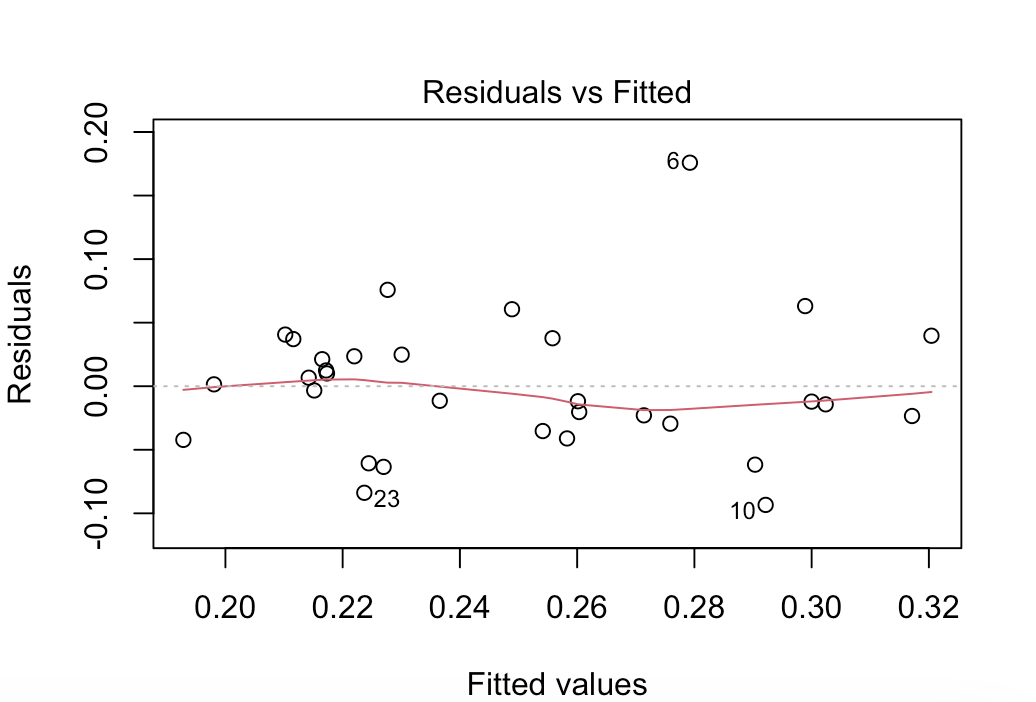 | 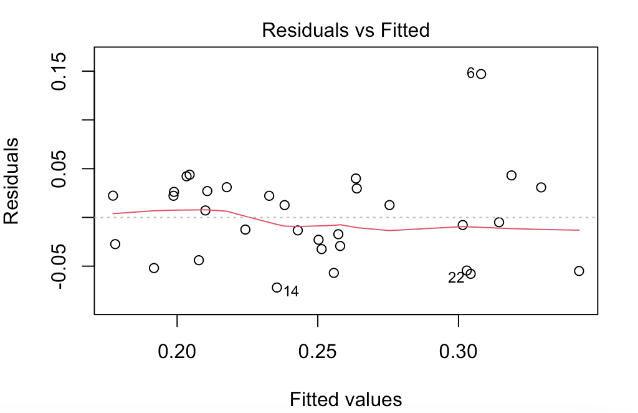 |

## **Placebo treatment:**

| **Model1:** | **Model 2:** |
| --- | --- |
| studentized Breusch-Pagan test  BP = 6.7512, df = 7, p-value = 0.4552 | studentized Breusch-Pagan test  BP = 7.2113, df = 13, p-value = 0.8909 |
| 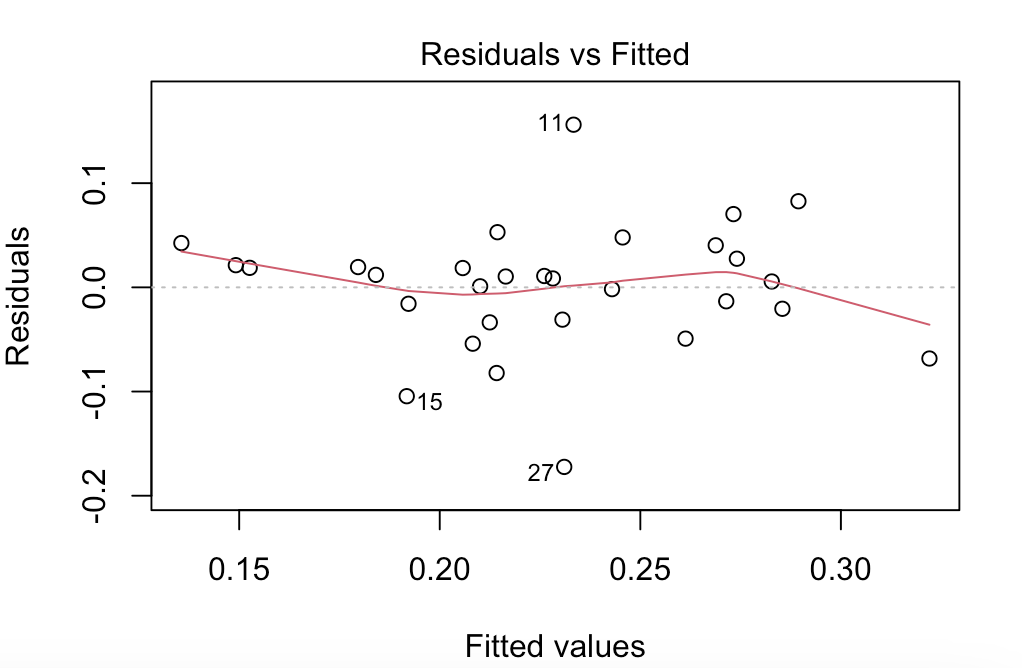 | 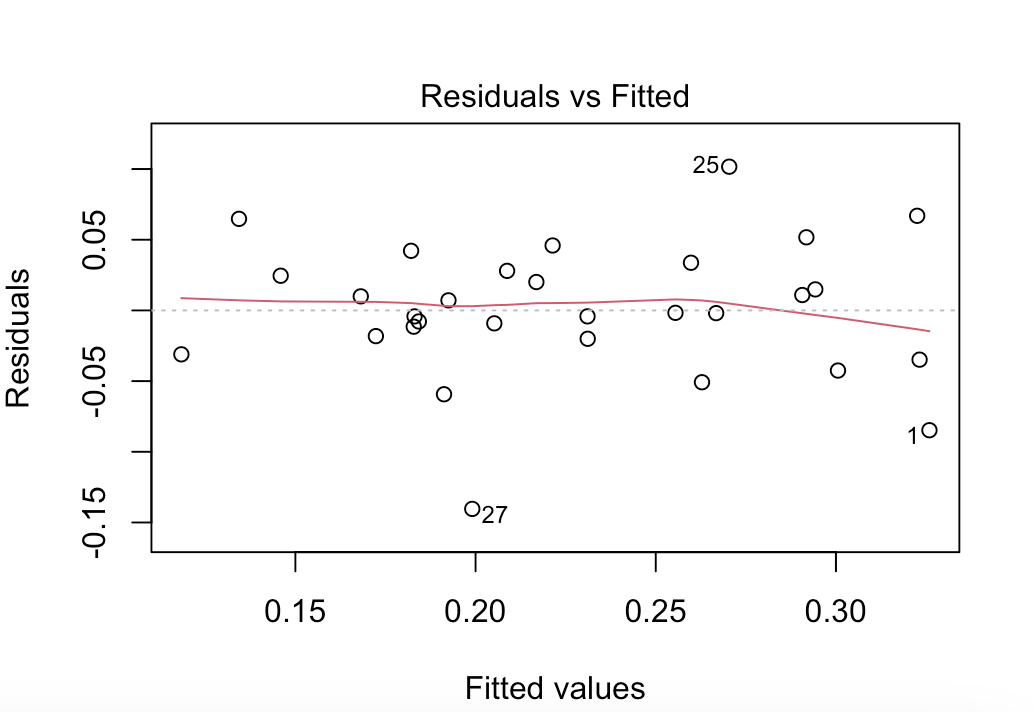 |

## **Test for normality**

## **Alutard treatment:**

| **Model1:** | **Model 2:** |
| --- | --- |
| Shapiro-Wilk normality test  W = 0.94372, p-value = 0.09549 | Shapiro-Wilk normality test  **W = 0.91506, p-value = 0.01531** |
| 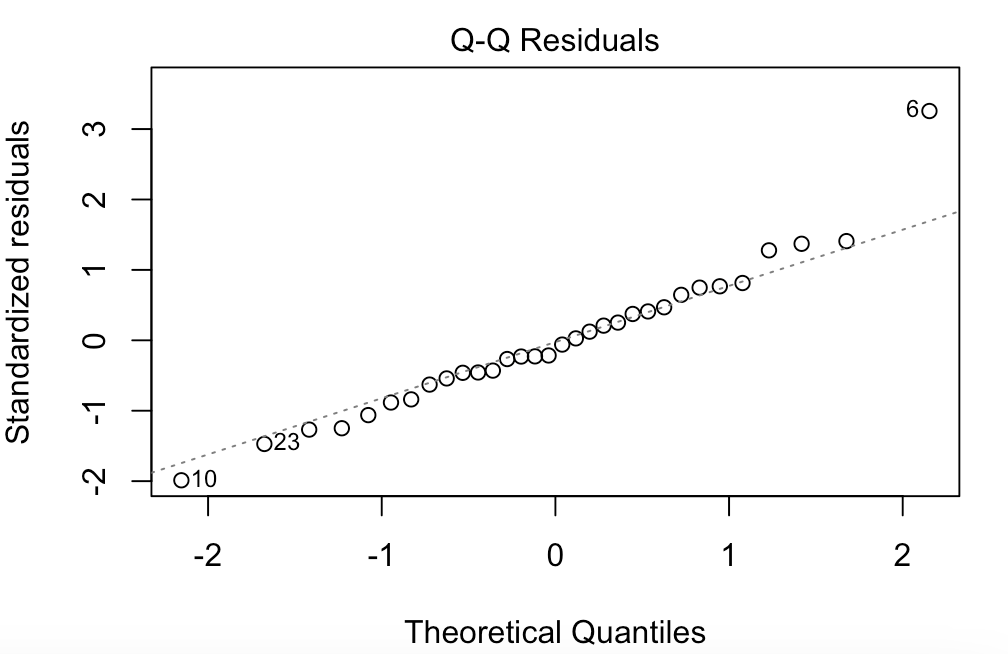 | **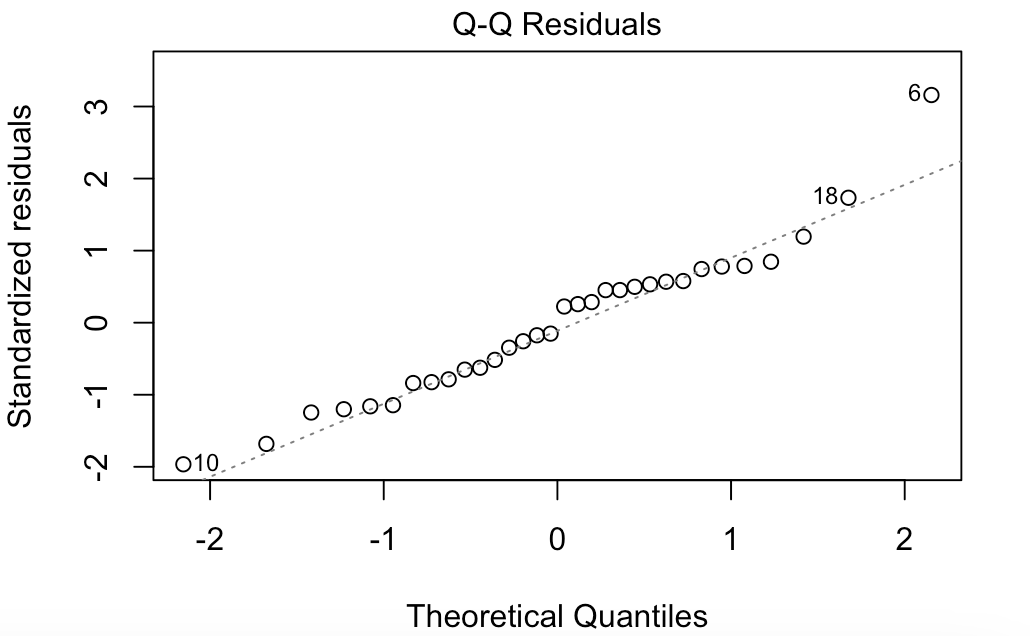** |

**Placebo treatment:**

| **Model1:** | **Model 2:** |
| --- | --- |
| Shapiro-Wilk normality test  W = 0.95577, p-value = 0.2406 | Shapiro-Wilk normality test  W = 0.96962, p-value = 0.5287 |
| 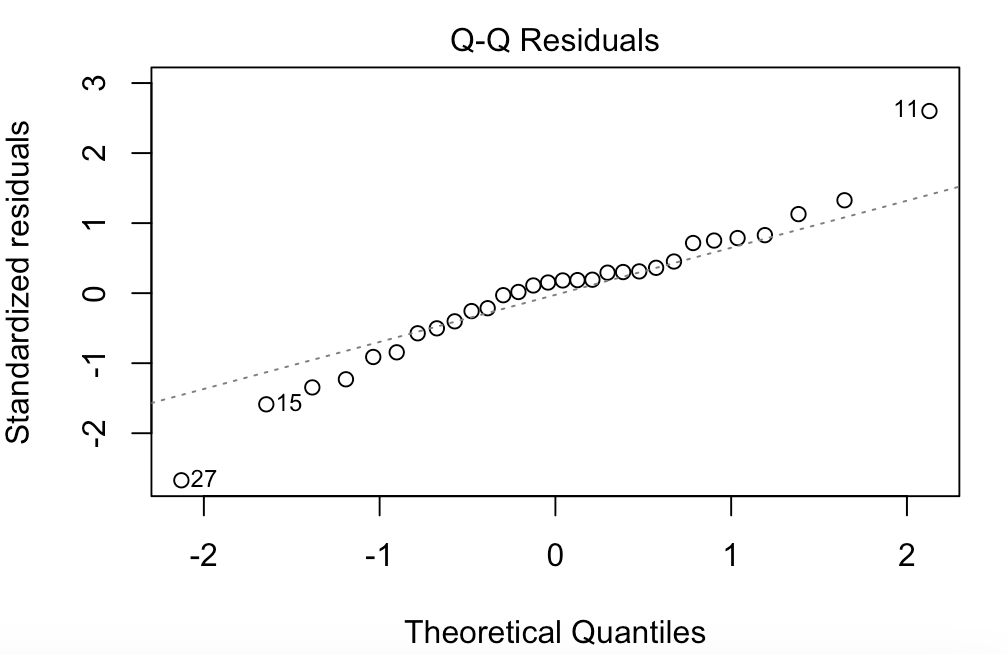 | 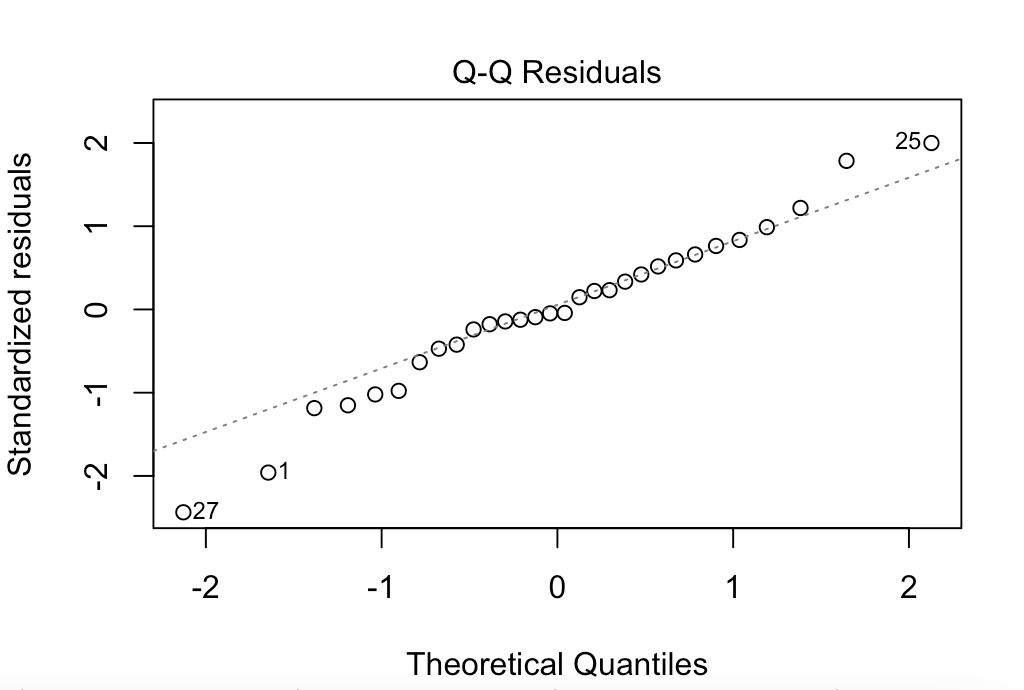 |

Since the test for normality was significant for model 2, including the timepoint, this model was rejected.

## **Luciferase reporter assay**

DNA isolated from H1299 lung cells was amplified using Phusion HSII polymerase (Thermo Scientific, Austria) with primers (Sigma, Austria, table below) containing the restriction enzyme sites at the 5´ends of the forward and reverse primer, respectively. The region of interest was amplified by using 1x GC buffer, 1% DMSO, 200 µM dNTPs, each 500 nM forward and reverse primer, 0.1 Unit Phusion HSII polymerase and 10 ng genomic DNA. The PCR protocol included an initial activation step at 98°C for 30 sec, followed by 45 cycles of 98°C for 10 sec, 67°C for 10 sec and 72°C for 15 sec, and a final elongation step at 72°C for 5 min. The PCR product was excised from the gel and cleaned up using the innuPREP DOUBLEpure kit (Innuscreen, Austria). In total, 500 ng of the amplicon and the plasmid were double digested with the restriction sites included in the primer sequences NsiI (NEB, Austria) and BamHI (NEB, Austria) at 37°C for 20 min. Insert and plasmid ligation was performed with a 1:5 plasmid to insert molecule ratio with T4 DNA ligase (NEB, Austria) at 16°C overnight. Transformation was performed by mixing 50 µl chemically competent *E. coli* GT115 cells (Invivogen, Belgium) with 5 µl of the ligation reaction mix and a 30 sec heat shock at 42°C. Transformation reactions were spread on LB-zeocin (50 µl/ml; Invivogen, Belgium) plates. After successful growth in larger volumes, the presence of the insert was verified by Sanger sequencing (LGC genomics; Germany, Supplement Method Section 1.1). Plasmids containing the desired inserts were extracted with the PureYield Plasmid Midiprep System (Promega, Austria). *In vitro* methylation of 3 µg constructs was performed with 8 U M.SssI (NEB, Austria) at 37°C for 3h. The enzyme was inactivated at 65°C for 20 min, followed by purification with the ReliaPrep DNA Clean-Up and Concentration kit (Promega, Austria).

H1299 lung cells were seeded into 24 well plates (0.5x10^5^ cells/well) with RPMI + 10% fetal bovine serum medium without antibiotics. The next day, 500 ng plasmid and 25 ng pGL4 SV40 (Promega, Austria) control plasmid were co-transfected with Lipofectamin 3000 (Thermo Scientific, Austria) and incubated overnight. After cell lysis, the lucia and firefly luciferase read-out was generated with a Tecan Spark plate reader (Tecan, Austria). The lucia activity was normalized to the firefly activity. To calculate the fold-change in gene expression, the empty vector was used as a control. Each experiment was performed in triplicates. Cells were regularly tested to exclude mycoplasma contaminations.

**Primer table:**

| **Primer** | **Primer-ID** | **Sequence 5´-3´** | **Amplicon length [bp]** |
| --- | --- | --- | --- |
| Forward | F-GCNT2-NsiI | tcagATGCATGTCCACCAAGAACTGTTAAACC | 303bp |
| Reverse | R-GCNT2-BamHI | tactGGATCCTTGACCATGCAGTGTTTATTCG |  |
| Forward | F-ABRA-NsiI | tcagATGCATTCCAGTTATCAAAGATCCTAAGTTG | 202bp |
| Reverse | R-ABRA-BamHI | tactGGATCCAGTTCAAAGTTGGGACTAGAAAG |  |
| Forward | Sequencing | GCAGATTAAAAGGAATTCCTGC |  |

# **Supplement Tables**

Supplement Table 1. Uniform manifold approximation and projection (UMAP) summary statistics. UMAP was performed on the beta value difference between post- and pre-treatment of the top 100 differentially methylated sites. SD, standard deviation. Mean ± standard deviation (SD) values are given for the first (UMAP1) and second (UMAP2) UMAP dimensions, calculated for the sample number (n).

| **Group** | **Mean UMAP1** | **Mean UMAP2** | **Mean UMAP3** | **SD UMAP1** | **SD**  **UMAP2** | **SD UMAP3** | **n** |
| --- | --- | --- | --- | --- | --- | --- | --- |
| Alutard | 0.04265413 | -0.01153790 | 0.4576721 | 0.7723663 | 10.210.940 | 0.5888011 | 16 |
| Placebo | -0.04549774 | 0.01230709 | -0.4881836 | 0.7685325 | 0.7307508 | 0.6486956 | 15 |

Supplement Table 2. Top differentially methylated sites in BPE-SCIT post- versus pre-treated samples with unadjusted *p*<0.00001Each row represents a single CpG site identified by its Illumina probe ID (CG ID), chromosomal location (Chr, Position) given in assembly hg38, and relation to a CpG island (CGI Relation; e.g., island, shore, shelf, open sea). The DNA methylation difference between groups is given as mean difference in beta value (Δβ). Significance is shown by the unadjusted *p*-value (unadj. *p*) and the false discovery rate-adjusted *p-*value (FDR adj. *p*). Associated genes (Gene) are annotated based on proximity to the CpG site. Sites with FDR adj. *p* < 0.05 were considered statistically significant.

| **No.** | **CG ID** | **Chr** | **Position** | **CGI Relation** | **Δβ** | **unadj. *p*** | **FDR**  **adj. *p*** | ***Gene*** |
| --- | --- | --- | --- | --- | --- | --- | --- | --- |
| 1 | cg22187251 | chr6 | 10529935 | Open Sea | 0.048 | 4.88E-09 | 0.00328 | GCNT2 |
| 2 | cg22834713 | chr6 | 28984341 | North Shore | -0.011 | 2.82E-08 | 0.00515 |  |
| 3 | cg24284822 | chr4 | 78432011 | Open Sea | -0.008 | 3.06E-08 | 0.00515 | CXCL13 |
| 4 | cg07417708 | chr5 | 78809348 | Island | 0.003 | 1.12E-07 | 0.01368 | HOMER1 |
| 5 | cg18380353 | chr14 | 53162676 | Island | 0.007 | 1.22E-07 | 0.01368 | ERO1A |
| 6 | cg11630696 | chr3 | 79817333 | South Shore | 0.013 | 1.88E-07 | 0.01807 | ROBO1 |
| 7 | cg13541429 | chr9 | 94711818 | Island | 0.014 | 2.39E-07 | 0.02009 | ROR2 |
| 8 | cg26140749 | chr8 | 143821266 | Island | -0.036 | 3.36E-07 | 0.02507 |  |
| 9 | cg09822136 | chr5 | 14144123 | Island | -0.008 | 3.84E-07 | 0.02531 | TRIO |
| 10 | cg06426689 | chr22 | 24572683 | Open Sea | 0.008 | 4.14E-07 | 0.02531 | SUSD2 |
| 11 | cg05365220 | chr1 | 94375159 | Island | -0.003 | 4.57E-07 | 0.02558 | GCLM |
| 12 | cg05735659 | chr12 | 48099394 | Island | -0.028 | 5.98E-07 | 0.02842 | RPAP3 |
| 13 | cg24504194 | chr6 | 31527920 | Open Sea | -0.028 | 5.98E-07 | 0.02842 |  |
| 14 | cg01340310 | chr17 | 536443 | Open Sea | -0.017 | 6.34E-07 | 0.02842 | VPS53 |
| 15 | cg19719340 | chr5 | 74162223 | Island | -0.000 | 6.89E-07 | 0.02897 | FAM169A |
| 16 | cg08798692 | chr6 | 33377958 | Island | -0.007 | 7.77E-07 | 0.03073 | PHF1 |
| 17 | cg16199665 | chr6 | 33168045 | Island | -0.025 | 1.08E-06 | 0.03941 | SLC39A7 |
| 18 | cg00054971 | chr10 | 129796937 | Open Sea | -0.018 | 1.11E-06 | 0.03941 | PTPRE |
| 19 | cg09207494 | chr14 | 65453864 | Island | -0.007 | 1.32E-06 | 0.04431 | NA |
| 20 | cg23782880 | chr6 | 28749781 | Open Sea | -0.009 | 1.55E-06 | 0.04954 | NA |
| 21 | cg22336863 | chr8 | 107783076 | Open Sea | 0.068 | 2.32E-06 | 0.06775 | ABRA |
| 22 | cg09222993 | chr1 | 144913803 | Open Sea | -0.015 | 2.41E-06 | 0.06775 | NBPF20 |
| 23 | cg05986052 | chr7 | 6212576 | South Shore | 0.033 | 2.57E-06 | 0.06775 | CYTH3 |
| 24 | cg09246520 | chr2 | 208575839 | North Shore | -0.010 | 2.62E-06 | 0.06775 | CCNYL1 |
| 25 | cg08015590 | chr7 | 2281924 | Island | 0.012 | 2.62E-06 | 0.06775 | NUDT1 |
| 26 | cg14823666 | chr10 | 70166317 | Island | -0.016 | 2.95E-06 | 0.07352 | RUFY2 |
| 27 | cg23413007 | chr8 | 145734497 | Island | -0.005 | 3.66E-06 | 0.08786 | MFSD3 |
| 28 | cg06333492 | chr15 | 65903461 | Island | 0.003 | 3.92E-06 | 0.08858 | SLC24A1 |
| 29 | cg08792185 | chr6 | 31855422 | North Shore | 0.019 | 3.95E-06 | 0.08858 | EHMT2 |
| 30 | cg25443689 | chr5 | 64858972 | Island | -0.011 | 4.32E-06 | 0.09369 | PPWD1 |
| 31 | cg11163975 | chr4 | 118006812 | Island | -0.022 | 4.55E-06 | 0.09553 | TRAM1L1 |
| 32 | cg04813639 | chr15 | 79383947 | Island | -0.003 | 4.97E-06 | 0.10117 | RASGRF1 |
| 33 | cg11592619 | chr2 | 239067608 | Island | -0.021 | 5.13E-06 | 0.10150 |  |
| 34 | cg02013527 | chr5 | 88426579 | Open Sea | 0.039 | 6.01E-06 | 0.11536 |  |
| 35 | cg17175324 | chr11 | 64009423 | Island | -0.008 | 6.78E-06 | 0.12270 | FKBP2 |
| 36 | cg10510742 | chr16 | 30620917 | North Shore | -0.035 | 6.85E-06 | 0.12270 | ZNF689 |
| 37 | cg24728105 | chr11 | 2418157 | South Shore | -0.004 | 6.93E-06 | 0.12270 | TSSC4 |
| 38 | cg07481320 | chr6 | 42739021 | Open Sea | 0.002 | 7.83E-06 | 0.13494 |  |
| 39 | cg17765553 | chr8 | 35731494 | Open Sea | -0.002 | 8.23E-06 | 0.13728 |  |
| 40 | cg19315948 | chr4 | 135352683 | Open Sea | 0.038 | 8.52E-06 | 0.13728 |  |
| 41 | cg05698732 | chr13 | 110816607 | Open Sea | 0.015 | 8.58E-06 | 0.13728 | COL4A1 |
| 42 | cg26175649 | chr6 | 34919755 | Open Sea | 0.013 | 8.86E-06 | 0.13861 | ANKS1A |
| 43 | cg09210323 | chr5 | 77409636 | Open Sea | 0.036 | 9.55E-06 | 0.14092 | AP3B1 |
| 44 | cg12926300 | chr5 | 143253949 | Open Sea | 0.024 | 9.57E-06 | 0.14092 |  |
| 45 | cg19839421 | chr17 | 27047098 | Island | -0.002 | 9.64E-06 | 0.14092 | RPL23A |

Supplement Table 3. Top differentially methylated sites in BPE-SCIT post- versus pre-treatment identified in the HLA gene complex on chromosome 6. Each row represents a single CpG site identified by its Illumina probe ID (CG ID) and the chromosomal location (Chr, Position) given in assembly hg38. The DNA methylation difference between groups is given as mean difference in beta value (Δβ). Significance is shown by the unadjusted *p*-value (unadj. *p*) and the false discovery rate–adjusted *p-*value (FDR adj. *p*). Associated genes (Gene) are annotated based on proximity to the CpG site. Sites with FDR adj. *p* < 0.05 were considered statistically significant.

| **No.** | **CG ID** | **Chr.** | **Position** | **Δβ** | **unadj*. p*** | **FDR**  **adj. *p*** |
| --- | --- | --- | --- | --- | --- | --- |
| **1** | cg22834713 | 6 | 28984341 | -0.011 | 2.82E-08 | 0.00515 |
| **2** | cg24504194 | 6 | 31527920 | -0.028 | 5.98E-07 | 0.02842 |
| **3** | cg08798692 | 6 | 33377958 | -0.007 | 7.77E-07 | 0.03073 |
| **4** | cg16199665 | 6 | 33168045 | -0.025 | 1.08E-06 | 0.03941 |
| **5** | cg23782880 | 6 | 28749781 | -0.009 | 1.55E-06 | 0.04954 |

**Supplement Table 4. Top differentially methylated sites in placebo post- versus pre-treated samples with unadjusted *p*<0.0001.** Each row represents a single CpG site identified by its Illumina probe ID (CG ID), chromosomal location (Chr, Position) given in assembly hg38, and relation to a CpG island (CGI Relation; e.g., island, shore, shelf, open sea. The DNA methylation difference between groups is given as mean difference in beta value (Δβ). Significance is shown by the unadjusted *p*-value (unadj. *p*) and the false discovery rate–adjusted *p-*value (FDR adj. *p*). Associated genes (Gene) are annotated based on proximity to the CpG site. Sites with FDR adj. *p* < 0.05 were considered statistically significant.

| **No.** | **CG ID** | **Chr** | **Position** | **CGI Relation** | **Δβ** | **unadj. *p*** | **FDR**  **adj. *p*** | ***Gene*** |
| --- | --- | --- | --- | --- | --- | --- | --- | --- |
| 1 | cg16906347 | chr11 | 75352202 | South Shore | 0.003 | 2.29E-06 | 0.67952 | MAP6 |
| 2 | cg02962380 | chr16 | 79600826 | Island | 0.000 | 3.07E-06 | 0.67952 |  |
| 3 | cg26756479 | chr1 | 150364488 | Island | 0.020 | 5.83E-06 | 0.67952 | RPRD2 |
| 4 | cg00635204 | chr14 | 72731705 | Open Sea | 0.045 | 6.81E-06 | 0.67952 | RGS6 |
| 5 | cg13267619 | chr3 | 36770510 | Open Sea | -0.010 | 7.23E-06 | 0.67952 | DCLK3 |
| 6 | cg07575409 | chr6 | 18694034 | Open Sea | 0.000 | 7.87E-06 | 0.67952 | NA |
| 7 | cg00729860 | chr11 | 31173907 | Open Sea | -0.017 | 8.93E-06 | 0.67952 | DCDC1 |
| 8 | cg02342092 | chr2 | 44087257 | Island | 0.006 | 1.03E-05 | 0.67952 | ABCG8 |
| 9 | cg22209752 | chr3 | 63864416 | Island | 0.003 | 1.04E-05 | 0.67952 | ATXN7 |
| 10 | cg08339189 | chr9 | 121498219 | North Shore | -0.011 | 1.08E-05 | 0.67952 |  |
| 11 | cg08839831 | chr2 | 195670828 | Open Sea | -0.010 | 1.11E-05 | 0.67952 |  |
| 12 | cg15207885 | chr7 | 73506154 | Open Sea | -0.010 | 1.29E-05 | 0.68580 | LIMK1 |
| 13 | cg00495512 | chr7 | 114843155 | Open Sea | -0.008 | 1.33E-05 | 0.68580 |  |
| 14 | cg03056811 | chr1 | 160670123 | Open Sea | -0.006 | 1.62E-05 | 0.70313 | CD48 |
| 15 | cg03853593 | chr5 | 157653892 | South Shore | -0.006 | 1.81E-05 | 0.70313 |  |
| 16 | cg21454227 | chr10 | 12068800 | Island | 0.003 | 1.86E-05 | 0.70313 | UPF2 |
| 17 | cg04031757 | chr6 | 28825552 | Open Sea | 0.006 | 2.04E-05 | 0.70313 |  |
| 18 | cg05962033 | chr1 | 5886268 | Open Sea | 0.016 | 2.09E-05 | 0.70313 |  |
| 19 | cg23856428 | chr8 | 130287981 | Open Sea | -0.004 | 2.17E-05 | 0.70313 |  |
| 20 | cg25698371 | chr9 | 96450059 | Island | 0.003 | 2.34E-05 | 0.70313 |  |
| 21 | cg08211413 | chr14 | 24316641 | Island | 0.043 | 2.34E-05 | 0.70313 |  |
| 22 | cg18832902 | chr6 | 30061666 | South Shore | -0.001 | 2.34E-05 | 0.70313 |  |
| 23 | cg05063944 | chr11 | 107859311 | South Shore | 0.001 | 2.44E-05 | 0.70313 |  |
| 24 | cg12965426 | chr2 | 131106142 | South Shore | 0.009 | 2.53E-05 | 0.70313 |  |
| 25 | cg27148609 | chr15 | 90755860 | Open Sea | -0.001 | 2.61E-05 | 0.70313 | SEMA4B |
| 26 | cg11107196 | chr16 | 71884362 | Island | -0.003 | 2.86E-05 | 0.71391 | ATXN1L |
| 27 | cg15485670 | chr19 | 45506983 | Island | 0.012 | 2.87E-05 | 0.71391 | RELB |
| 28 | cg19130313 | chr6 | 21078710 | Open Sea | -0.007 | 3.14E-05 | 0.71928 | CDKAL1 |
| 29 | cg11558551 | chr19 | 17405633 | Open Sea | 0.000 | 3.18E-05 | 0.71928 | ABHD8 |
| 30 | cg18015844 | chr6 | 33451869 | North Shelf | 0.000 | 3.28E-05 | 0.71928 |  |
| 31 | cg01622379 | chr1 | 75128316 | North Shore | 0.013 | 3.32E-05 | 0.71928 | ERICH3 |
| 32 | cg08384379 | chr14 | 73197635 | Open Sea | -0.003 | 3.55E-05 | 0.74530 | DPF3 |
| 33 | cg03548242 | chr1 | 108693068 | South Shore | 0.011 | 3.80E-05 | 0.77510 | SLC25A24 |
| 34 | cg10131257 | chr4 | 73452727 | Open Sea | -0.006 | 4.07E-05 | 0.80483 |  |
| 35 | cg25589352 | chr8 | 109975939 | South Shore | 0.013 | 4.42E-05 | 0.82091 |  |
| 36 | cg17685905 | chr14 | 80568703 | Open Sea | -0.011 | 4.46E-05 | 0.82091 |  |
| 37 | cg05678157 | chr10 | 72525441 | Open Sea | -0.005 | 4.52E-05 | 0.82091 |  |
| 38 | cg11147301 | chr2 | 110120859 | Open Sea | -0.010 | 4.71E-05 | 0.82368 | SH3RF3 |
| 39 | cg06730002 | chr3 | 45865364 | Open Sea | 0.080 | 4.80E-05 | 0.82368 | LZTFL1 |
| 40 | cg11041876 | chr15 | 90911838 | Open Sea | -0.005 | 5.00E-05 | 0.82368 |  |
| 41 | cg20587400 | chr18 | 74212742 | Open Sea | -0.004 | 5.22E-05 | 0.82368 |  |
| 42 | cg04172501 | chr1 | 1201050 | Island | -0.003 | 5.33E-05 | 0.82368 | UBE2J2 |
| 43 | cg15066954 | chr5 | 161546931 | North Shore | -0.001 | 5.33E-05 | 0.82368 | GABRG2 |
| 44 | cg26302190 | chr2 | 146531948 | Open Sea | -0.008 | 5.50E-05 | 0.82368 |  |
| 45 | cg09125063 | chr17 | 4163812 | Open Sea | -0.013 | 5.51E-05 | 0.82368 | ANKFY1 |
| 46 | cg13010046 | chr11 | 34191375 | Open Sea | 0.023 | 5.84E-05 | 0.85306 | ABTB2 |
| 47 | cg13202845 | chr6 | 142089079 | South Shore | -0.016 | 5.96E-05 | 0.85306 | NA |
| 48 | cg13869971 | chr19 | 35545226 | Island | 0.006 | 6.72E-05 | 0.86011 | HPN |
| 49 | cg18393173 | chr6 | 34888358 | North Shore | 0.002 | 6.77E-05 | 0.86011 | ANKS1A |
| 50 | cg13867248 | chr6 | 33317807 | Island | -0.002 | 6.85E-05 | 0.86011 |  |
| 51 | cg06140118 | chr16 | 68022875 | Island | 0.001 | 6.89E-05 | 0.86011 | DPEP2 |
| 52 | cg08256298 | chr12 | 32366005 | Open Sea | -0.009 | 6.93E-05 | 0.86011 | BICD1 |
| 53 | cg02151463 | chr7 | 109960342 | Open Sea | 0.015 | 6.97E-05 | 0.86011 |  |
| 54 | cg03832371 | chr10 | 7248583 | Open Sea | -0.001 | 7.00E-05 | 0.86011 | SFMBT2 |
| 55 | cg12366597 | chr12 | 58913740 | Open Sea | -0.003 | 7.04E-05 | 0.86011 |  |
| 56 | cg22730029 | chr13 | 41019446 | Open Sea | 0.005 | 7.87E-05 | 0.86195 | LINC00598 |
| 57 | cg19810092 | chr6 | 89632688 | Open Sea | -0.001 | 7.90E-05 | 0.86195 | RNGTT |
| 58 | cg06131143 | chr2 | 48530473 | Island | -0.003 | 7.91E-05 | 0.86195 |  |
| 59 | cg15730879 | chr1 | 190098923 | Open Sea | 0.001 | 7.97E-05 | 0.86195 | BRINP3 |
| 60 | cg10003961 | chr6 | 167893069 | Open Sea | 0.071 | 8.06E-05 | 0.86195 |  |
| 61 | cg04510049 | chr5 | 178573773 | North Shelf | -0.009 | 8.08E-05 | 0.86195 | ADAMTS2 |
| 62 | cg26440287 | chr10 | 122411497 | Open Sea | -0.004 | 8.15E-05 | 0.86195 |  |
| 63 | cg09033731 | chr1 | 53941574 | North Shelf | 0.002 | 8.50E-05 | 0.86195 |  |
| 64 | cg15425427 | chr11 | 65638683 | North Shelf | 0.004 | 8.97E-05 | 0.86195 | EFEMP2 |
| 65 | cg23978557 | chr6 | 109439616 | North Shore | 0.002 | 8.98E-05 | 0.86195 | CEP57L1 |
| 66 | cg19081004 | chr5 | 6752403 | North Shelf | 0.021 | 9.30E-05 | 0.86195 | TENT4A |
| 67 | cg00785632 | chr2 | 170013922 | Open Sea | -0.011 | 9.33E-05 | 0.86195 | LRP2 |
| 68 | cg15821792 | chr4 | 127880521 | North Shore | 0.005 | 9.34E-05 | 0.86195 |  |
| 69 | cg14526804 | chr20 | 63156331 | North Shore | 0.003 | 9.69E-05 | 0.86195 |  |
| 70 | cg05723277 | chr11 | 44351029 | Open Sea | 0.001 | 9.71E-05 | 0.86195 |  |
| 71 | cg10948471 | chr3 | 185285202 | South Shelf | -0.035 | 9.72E-05 | 0.86195 |  |
| 72 | cg16362014 | chr5 | 118985861 | North Shelf | -0.007 | 9.75E-05 | 0.86195 |  |

# **Supplement Figures**


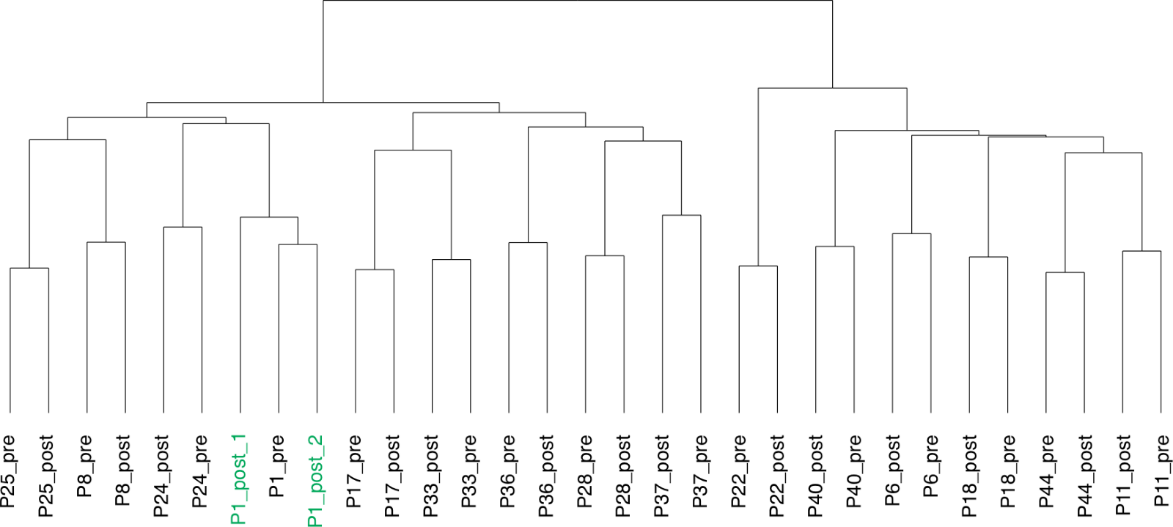


**Supplement Figure 1.** **Sample clustering using Manhattan distance of placebo post- and pre-treatment.** Replicates for placebo P1 (green) cluster in one branch, indicating reproducibility of the two batches.

**Supplement Figure 2.** **Principle component analysis (PCA) based on the difference in beta values between pre- and post-treatment of the top 100 differentially methylated probes (DMPs)**. Since Kaiser-Meyer-Olkin (KMO) measure of sampling adequacy and Bartlett’s test of sphericity were not successful, we have chosen to use the UMAP analysis, a non-linear dimensionality reduction technique, instead. The difference in distribution between the two groups was calculated between Q2 and Q3 using a two-tailed Mann Whitney test, **** *p*<0.0001.


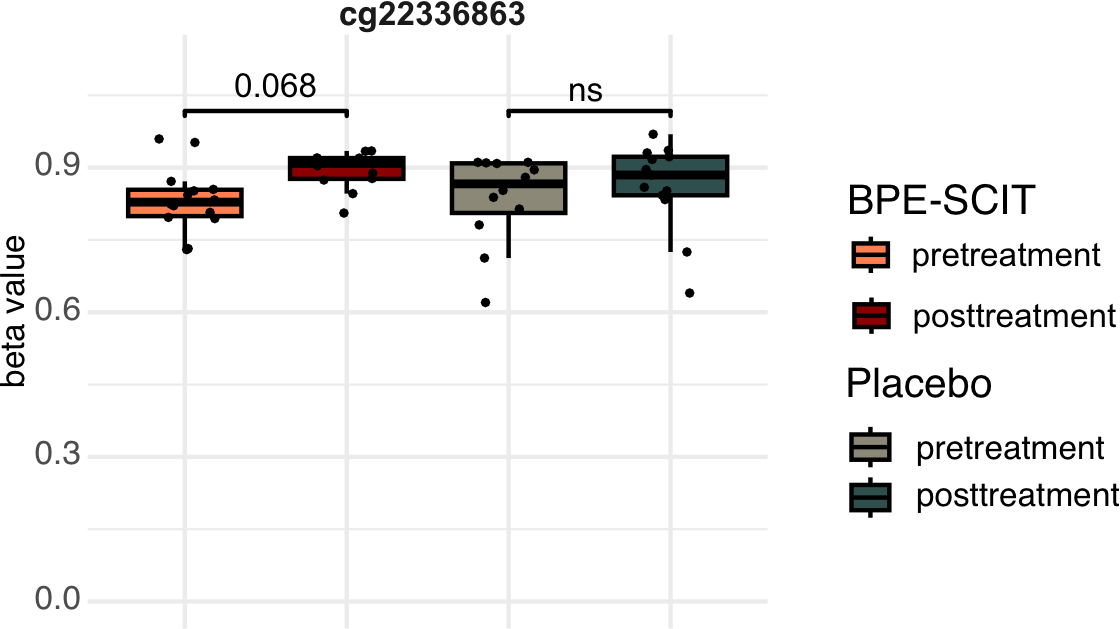


Supplement Figure 3. DNA methylation in *ABRA* cg22336863 in BPE-SCIT and placebo post- and pre-treatment. Barely significant changes were detected for pre- versus post- treated BPE-SCIT group, while no significant change was obtained for the placebo treated group. FDR corrected *p*-values from limma (6).


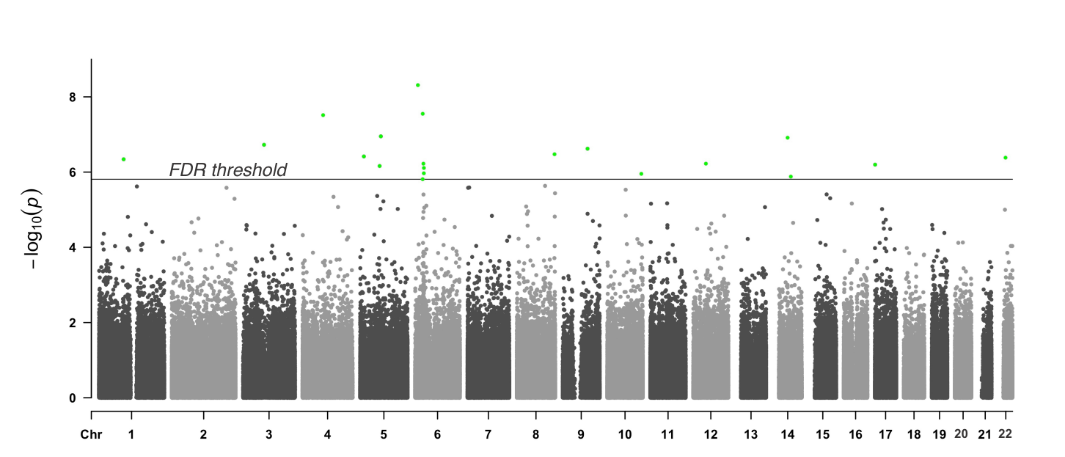


Supplement Figure 4. Manhattan plot of post- versus pre-treatment with BPE-SCIT using log unadjusted *p* values. Chr. 6 showed several CpG positions reaching statistical significance, but only a minor difference in DNA methylation was observed for these (see Supplement Table 3) and many others (see Supplement Table 1).


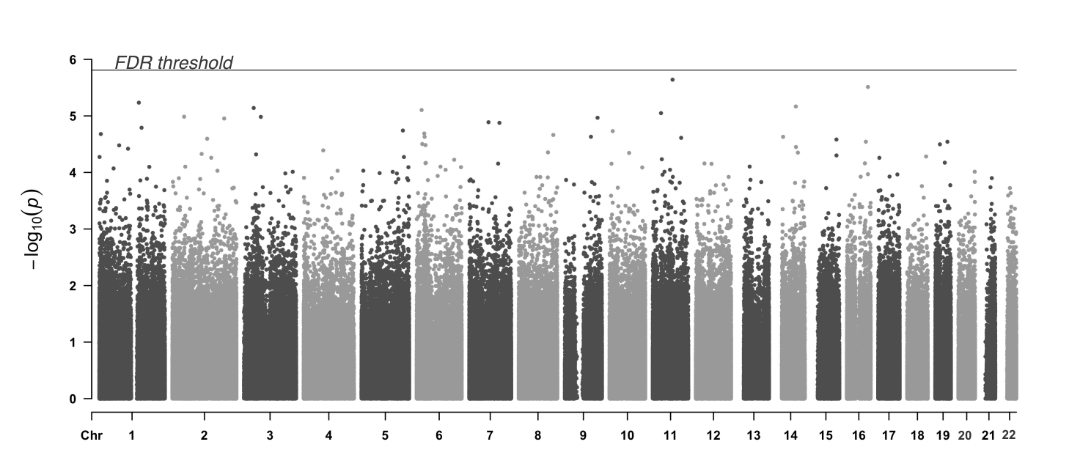


Supplement Figure 5. Manhattan plot of post- versus pre-treatment with placebo using log unadjusted *p* values. For placebo treatment no significantly differentially methylated positions were detected.


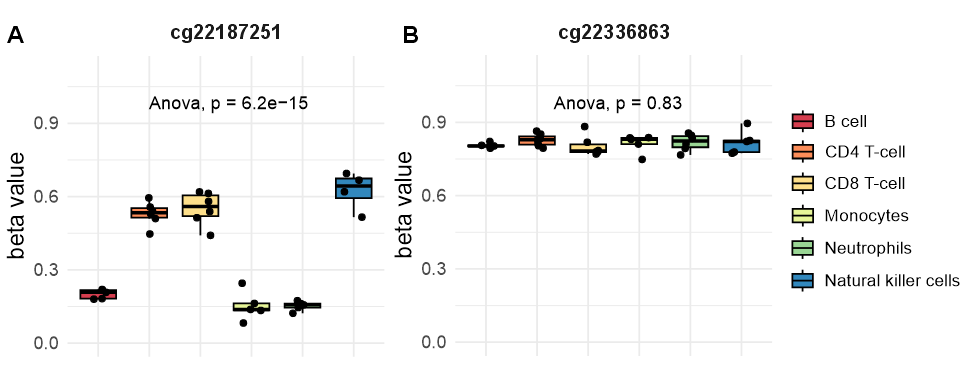


Supplement Figure 6. DNA methylation of A. *GCNT2* cg22187251 and B. *ABRA* cg22336863 in different cell types. While DNA methylation (beta value) varies significantly in different cell types for cg22187251, this is not the case for cg22336863 (Anova),


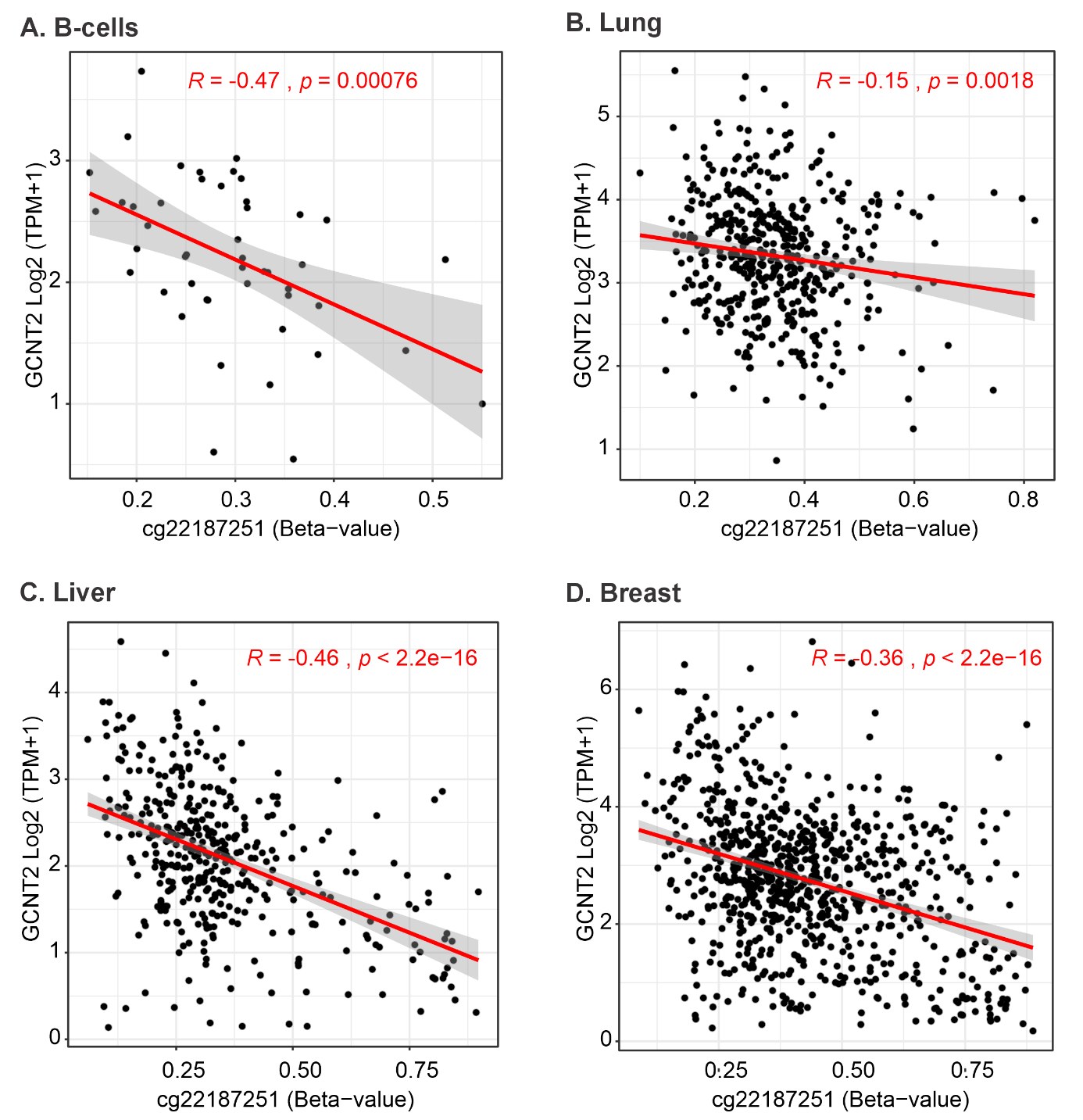


Supplement Figure 7. Correlation of DNA methylation at cg22187251 (promoter region) with expression of *GCNT2* in different cell types and tissues. Significant negative correlation of GCNT2 gene expression with DNA methylation (beta value) at position cg22187251 is shown for A. B-cells, B. lung , C. liver and D. breast tissue obtained from *The Cancer Genome Atlas* (TCGA, (12)) and visualized with Shiny Methylation Analysis Resource Tool (SMART) (13). Statistical analysis was performed using Pearson correlation with mean aggregation.

Supplement Figure 8. GCNT2 ELISA standard curve (left) and results of whole blood samples (right). All whole blood samples were below the detection limit and no significant difference between the groups was observed using a paired t-test for intra-group and an unpaired t-test for inter-group comparison.


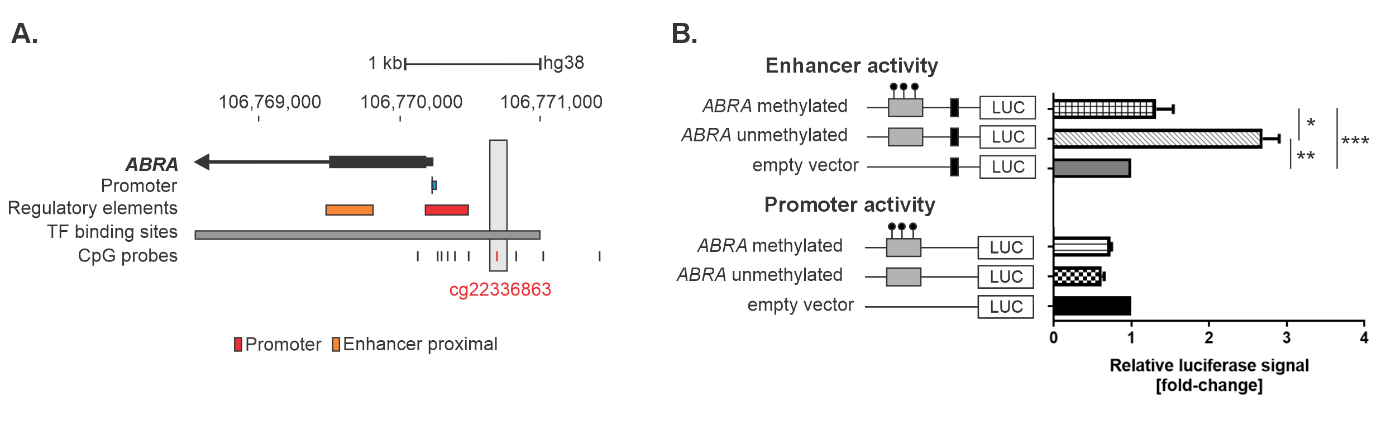


**Supplement Figure 9. *ABRA* cg22336863 harbors regulatory functions. A.** Shows the genomic annotation, promoters, regulatory elements and transcription factor (TF) binding regions upstream region of ABRA. Furthermore, CpG probe locations from the Infinium MethylationEPIC array are depicted. The grey highlighted region was analyzed in a luciferase reporter assay. **B.** Luciferase reporter assays using CpGfree vectors revealed an enhancer function of the region containing cg22336863, which was reduced when the insert was methylated. Pairwise comparisons using Welch two sample t-test; * *p*<0.05, ** *p*<0.01, *** *p*<0.001

**Supplement Figure 10. Example of Spearman correlation for cg01993027-SRSF4 with Bet v 1 sIgG_4_ (A.), sIgG (C.), and sIgG_4_/sIgG_1_ ratio (E.), as well as the BPE sIgG_4_ (B.), sIgG (D.), and sIgG_4_/sIgG_1_ ratio (F.) values.** * *p*<0.05, ** *p*<0.01

**Supplement Figure 11. DNA methylation of cg01993027-SRSF4, cg19898963-LRRC7, cg21436032, cg26856604-KIF1B and cg25532922-LBH correlating significantly with ≥4 and ≤6 clinical biomarkers for AIT efficacy.** DNA methylation (beta values) are shown. For this explorative analysis the non-adjusted p-values have been used. Statistical significance was assessed using a paired t-test for intra-group and an unpaired t-test for inter-group comparison. * *p*<0.05, ** *p*<0.01


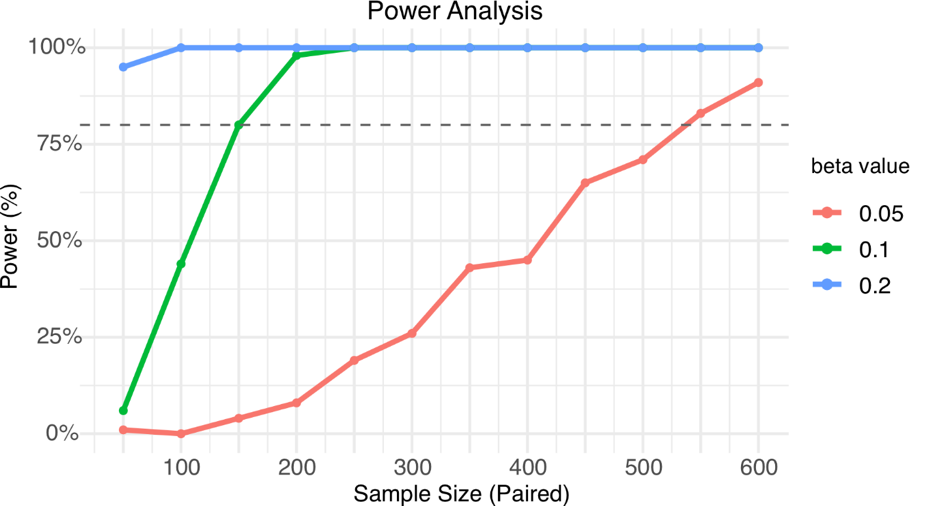


**Supplement Figure 12. Power Analysis.** To detect DNA methylation changes with a mean difference in beta value (Δβ) of 0.05, 0.1 or 0.2 with a power of 80% and *p*<1e-6, at least 537.8, 143.3 and 44.2 sample pairs are required. This calculation was performed with the R package pwr.t.test using a paired t-test.

# **References**

1. Teschendorff AE, Marabita F, Lechner M, Bartlett T, Tegner J, Gomez-Cabrero D, et al. A beta-mixture quantile normalization method for correcting probe design bias in Illumina Infinium 450 k DNA methylation data. Bioinformatics. 2013;29(2):189-96.

2. Leek JT, Storey JD. Capturing heterogeneity in gene expression studies by surrogate variable analysis. PLoS Genet. 2007;3(9):1724-35.

3. Houseman EA, Accomando WP, Koestler DC, Christensen BC, Marsit CJ, Nelson HH, et al. DNA methylation arrays as surrogate measures of cell mixture distribution. BMC Bioinformatics. 2012;13:86.

4. Salas LA, Koestler DC, Butler RA, Hansen HM, Wiencke JK, Kelsey KT, et al. An optimized library for reference-based deconvolution of whole-blood biospecimens assayed using the Illumina HumanMethylationEPIC BeadArray. Genome Biol. 2018;19(1):64.

5. Muller F, Scherer M, Assenov Y, Lutsik P, Walter J, Lengauer T, et al. RnBeads 2.0: comprehensive analysis of DNA methylation data. Genome Biol. 2019;20(1):55.

6. Ritchie ME, Phipson B, Wu D, Hu Y, Law CW, Shi W, et al. limma powers differential expression analyses for RNA-sequencing and microarray studies. Nucleic Acids Res. 2015;43(7):e47.

7. Dedeurwaerder S, Defrance M, Calonne E, Denis H, Sotiriou C, Fuks F. Evaluation of the Infinium Methylation 450K technology. Epigenomics. 2011;3(6):771-84.

8. McInnes L, Healy J, Melville J. UMAP: Uniform Manifold Approximation and Projection for Dimension Reduction. . 2018.

9. Castellano-Escuder P, Zachman DK, Han K, Hirschey MD. Interpretable multi-omics integration with UMAP embeddings and density-based clustering. bioRxiv. 2024.

10. Stevens MHH, Oksanen J. Permutational Multivariate Analysis of Variance Using Distance Matrices. <https://search.r-project.org/CRAN/refmans/vegan/html/adonis.html>.

11. Glass ER, Dozmorov MG. Improving sensitivity of linear regression-based cell type-specific differential expression deconvolution with per-gene vs. global significance threshold. BMC Bioinformatics. 2016;17(Suppl 13):334.

12. Cancer Genome Atlas Research N, Weinstein JN, Collisson EA, Mills GB, Shaw KR, Ozenberger BA, et al. The Cancer Genome Atlas Pan-Cancer analysis project. Nat Genet. 2013;45(10):1113-20.

13. Li Y, Ge D, Lu C. The SMART App: an interactive web application for comprehensive DNA methylation analysis and visualization. Epigenetics Chromatin. 2019;12(1):71.
